# Integrative genomic study of Chinese clear cell renal cell carcinoma reveals features associated with thrombus

**DOI:** 10.1038/s41467-020-14601-9

**Published:** 2020-02-06

**Authors:** Xiang-Ming Wang, Yang Lu, Yi-Meng Song, Jun Dong, Ruo-Yan Li, Guo-Liang Wang, Xu Wang, Shu-Dong Zhang, Zhou-Huan Dong, Min Lu, Shi-Yu Wang, Li-Yuan Ge, Guang-Da Luo, Run-Zhuo Ma, Steve George Rozen, Fan Bai, Di Wu, Lu-Lin Ma

**Affiliations:** 1 Biomedical Pioneering Innovation Center (BIOPIC) & Department of Urology, School of Life Sciences, Third Hospital, Peking University, Beijing, China; 2 Department of Nephrology, Chinese PLA General Hospital, Chinese PLA Institute of Nephrology, State Key Laboratory of Kidney Diseases, National Clinical Research Center for Kidney Diseases, Beijing, China; 3 Department of Urology, Chinese PLA General Hospital, Beijing, China; 4 Department of Pathology, Peking University Third Hospital, School of Basic Medical Sciences, Peking University Health Science Center, Beijing, China; 5 Centre for Computational Biology, Duke-NUS Medical School, Singapore, Singapore

**Keywords:** Cancer genomics, Renal cell carcinoma

## Abstract

Clear cell renal cell carcinoma (ccRCC) is a heterogeneous disease with features that vary by ethnicity. A systematic characterization of the genomic landscape of Chinese ccRCC is lacking, and features of ccRCC associated with tumor thrombus (ccRCC-TT) remain poorly understood. Here, we applied whole-exome sequencing on 110 normal-tumor pairs and 42 normal-tumor-thrombus triples, and transcriptome sequencing on 61 tumor-normal pairs and 30 primary-thrombus pairs from 152 Chinese patients with ccRCC. Our analysis reveals that a mutational signature associated with aristolochic acid (AA) exposure is widespread in Chinese ccRCC. Tumors from patients with ccRCC-TT show a higher mutational burden and genomic instability; in addition, mutations in *BAP1* and *SETD2* are highly enriched in patients with ccRCC-TT. Moreover, patients with/without TT show distinct molecular characteristics. We reported the integrative genomic sequencing of Chinese ccRCC and identified the features associated with tumor thrombus, which may facilitate ccRCC diagnosis, prognosis and treatment.

## Abstract (web summary)

The genomic heterogeneity of clear cell renal cell carcinoma (ccRCC) across populations is poorly understood. Here, the authors analyse a cohort of Chinese ccRCC cases revealing a mutational signature associated with aristolochic acid exposure, and higher mutational burden and enrichment for *BAP1* and *SETD2* mutations in ccRCC cases associated with tumor thrombus.

## Introduction

Renal cell carcinoma (RCC) is a frequently diagnosed cancer originating from the renal epithelium, with an estimated 403,262 new incidences and 175,098 deaths globally in 2018 (ref.^1^). RCC encompasses a heterogeneous group of chemotherapy-resistant cancers with >10 histological and molecular subtypes, of which clear cell RCC (ccRCC) is most common and accounts for ~75% of RCC cases^2^. Systematic characterizations of the genomic landscape of RCC have been mainly conducted in ccRCC and in patients from Western countries^3–5^. In the Caucasian population, ccRCC is featured by ubiquitous biallelic inactivation of *VHL*, which can be caused by chromosome 3p loss, concomitant *VHL* mutation, or promoter methylation. Other frequent genomic alterations of ccRCC include mutations in chromatin and histone modifier genes such as *PBRM1*, *BAP1*, and *SETD2* (refs.^3–5^). However, little is known regarding the genomic landscape of Chinese ccRCC and how it is different from Western cohorts. Thus, there is an urgent need for genome-wide molecular profiling of Chinese ccRCC to elucidate potential differences linked to ethnicity, which may have important consequences for prognosis and treatment.

A unique clinical aspect of ccRCC is its ability to grow into the renal vein or inferior vena cava and form a tumor thrombus (TT). The venous thrombus is present in ~15% of ccRCC patients^6^. The prognosis for patients with a TT is poor if left untreated, with a median survival of 5 months and a 1-year disease-specific survival rate of only 29%^7^. Although advances in surgical management have improved the 5-year survival rate of ccRCC-TT patients^8^, high perioperative mortality, and postoperative complications are significant challenges. Furthermore, genomic studies of ccRCC-TT are very limited. Therefore, exploring the genomic features of ccRCC-TT and portraying the evolutionary process, leading from the primary tumor to the development of a TT are critically important.

In the current study, we report the results of our genomic and transcriptomic profiling of Chinese ccRCC. By comparing our data with a ccRCC data set from Western patients (The Cancer Genome Atlas Research Network, TCGA), we evaluate the similarities and differences between ccRCC in Chinese and Western patients. Of note, a mutational signature (MS) associated with aristolochic acid (AA) exposure is widely observed in Chinese patients^9^. Importantly, we find that inactivation of one of the chromatin remodeling genes *BAP1* and *SETD2* is significantly more common in ccRCC patients with TT. Our findings shed light on the molecular characteristics of Chinese ccRCC and reveal distinct genomic and transcriptomic features associated with TT, providing valuable biological and clinical insights into the disease.

## Results

### The mutational landscape of Chinese ccRCC

**Fig. 1:**
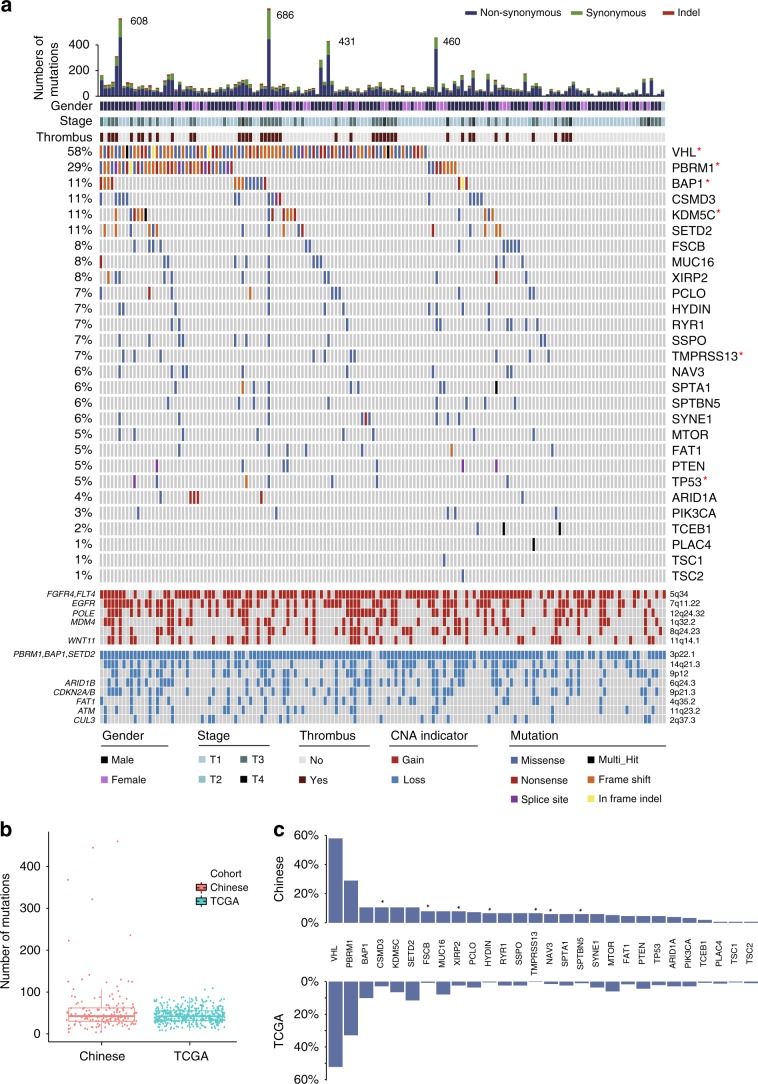
Landscape of mutations and copy number alterations of Chinese ccRCC. **a** Alteration landscape of 152 Chinese ccRCC primary tumors. Top histogram, the number of silent and non-slient mutations in each sample. Upper heat map, gender, thrombus, and tumor stage information. Middle heat map, distribution of ccRCC-associated cancer genes and top 15 genes across samples, with genes ranked by mutation frequency. Bottom heat map, copy number gains (red) and losses (blue), with potential driver genes encompassed by the cytobands shown on the left. SMG genes are marked by a red asterisk. **b** The nonsynonymous mutational burdens of the Chinese and TCGA cohorts were compared. The box plot displays the first and third quartiles (top and bottom of the boxes), the median (band inside the boxes), and the lowest and highest point within 1.5 times the interquartile range of the lower and higher quartile (whiskers). Wilcoxon rank-sum test, *p < 0.05, ** *p* < 0.01, ***p < 0.001. **c** The mutation frequencies of genes in the Chinese cohort and TCGA cohort. The gene list is derived from **a**. Genes with significantly different mutation rates between two cohorts are marked by black asterisks. Fisher’s exact test, *p *<* 0.05, **p *<* 0.01, ***p *<* 0.001. The source data underlying Fig.  1a–c are provided as a Source Data file.

The global landscape of somatic alterations in Chinese ccRCC generated from whole-exome sequencing (WES) data of 152 primary tumors (Supplementary Table 1, Supplementary Data 1 and 2, using the WES of 152 matched blood or normal tissues as controls) is shown in Fig. 1a. A total of 12,534 somatic changes were identified, including 12,012 single-nucleotide variants (SNVs) and 522 insertions or deletions (InDels). Overall, we observed comparable mutation rates between the Chinese and the TCGA cohorts (Median_Chinese_ = 42.5, Median_TCGA_ = 42, Fig. 1b). Six significantly mutated genes (SMGs) were identified in the Chinese cohort by MutSigCV^10^. In line with previous results from the TCGA study, *VHL*, *PBRM1*, *BAP1*, *TP53,* and *KDM5C* were identified as SMGs. However, *TMPRSS13* was identified as a SMG in the Chinese ccRCC cohort, but not in the Western cohort^3^.

Driver genes reported in previous ccRCC studies^3,4,11^ and the top 15 frequently mutated genes in our cohort are listed in Fig.  1a. Notably, *VHL* mutation was the most prominent variation (58%), followed by *PBRM1* (29%), *CSMD3* (11%), *BAP1* (11%), *SETD2* (11%), and *KDM5C* (11%). The mutation frequencies of most putative ccRCC driver genes were similar in Chinese and TCGA ccRCC patients (Fig. 1c). However, the Chinese cohort had significantly higher mutation frequencies in *CSMD3* (11% vs. 3%, Fisher’s exact test, *p* = 5.99e^−04^) and *TMPRSS13* (7% vs. 0.2%, Fisher’s exact test, *p* = 1.25e^−05^) in comparison with those of the TCGA cohort (Fig. 1c). *CSMD3* is a transmembrane receptor, and its homology member, *CSMD1*, is a putative suppressor of squamous cell carcinomas^12^. Moreover, recent studies showed that loss of *CSMD3* could increase the proliferation of airway epithelial cells and is involved in the tumorigenesis of lung cancer^13^.

To investigate somatic copy number alterations (SCNAs) in Chinese ccRCC, GISTIC analysis^14^ was used to identify recurrent SCNA regions. Consistent with the TCGA cohort, our data showed that the most frequent arm-level events were chromosome 3p loss and 5q gain (Fig. 1a and Supplementary Fig.  1). Focal amplifications involved some oncogenes, such as *EGFR* at 7q11.22, *MDM4* at 1q32.2, *POLE* at 12q24.32, and *WNT11* at 11q14.1. Focally deleted regions included the tumor suppressor genes *ATM* at 11q23.2, *CUL3* at 2q37.3, *ARID1B* at 6q24.3, *FAT1* at 4q35.2 and *CDKN2A* at 9p21.3 (Fig. 1a). Some new recurrent SCNA regions were identified in Chinese ccRCC, including 7q11.22 gain, 11q14.1 gain, 12q24.32 gain, and 6q24.3 loss (Fig. 1a).

### Enriched AA signature in Chinese ccRCC

**Fig. 2:**
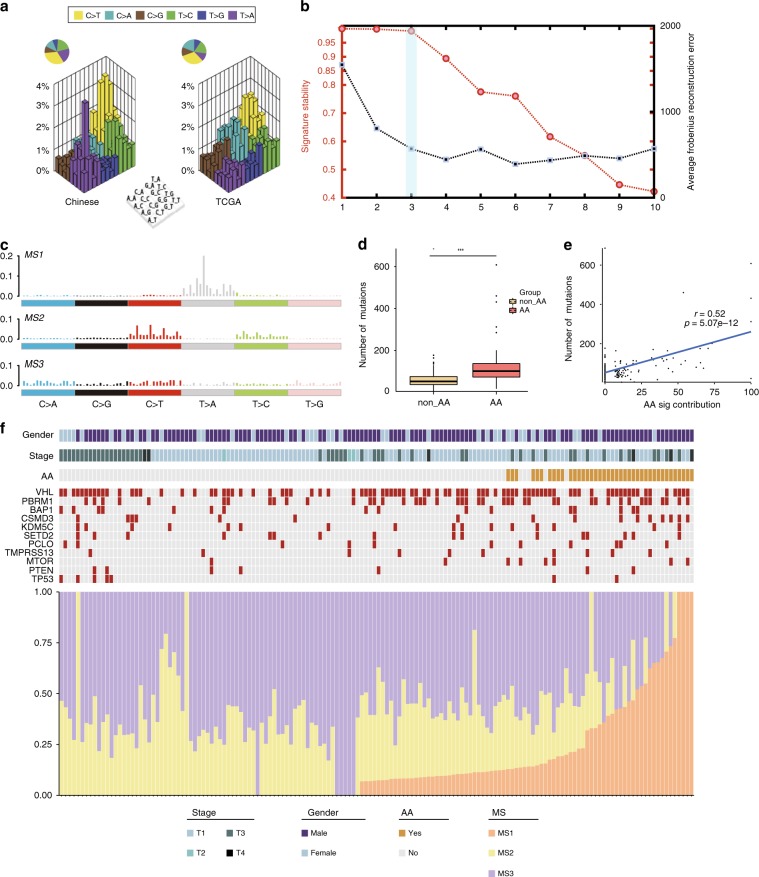
Inferred mutational signatures and their contributions in Chinese ccRCC patients. **a** ‘Lego’ plots display the frequency of 96 subtypes of base substitutions in Chinese ccRCC (left) and TCGA ccRCC (right). **b** Identifying the number of processes operating in a set of 152 ccRCC samples based on the reproducibility of their signatures and average Frobenius reconstruction error. **c** Three mutational signatures deciphered from the base substitutions identified in 152 ccRCC genomes. **d** The mutational burden was associated with the AA signature, and patients in the AA signature group had a heavier mutation load. The box plot displays the first and third quartiles (top and bottom of the boxes), the median (band inside the boxes), and the lowest and highest point within 1.5 times the interquartile range of the lower and higher quartile (whiskers). Wilcoxon rank-sum test, *p *<* 0.05, **p *<* 0.01, ***p *<* 0.001. **e** The somatic mutation load was positively associated with the contribution of the AA signature (Pearson’s correlation coefficient, two-tailed *t* test). **f** Contributions of each mutational signature per sample. The upper heat map shows sample gender information, tumor stage, mSigAct results, and the mutation landscape of 11 genes. The source data underlying Figs. 2c–f are provided as a Source Data file.

To explore the specific etiological factors that may contribute to the mutagenesis of Chinese ccRCC, we first compared the mutational spectra of Chinese and Western cohorts. The T > A transversion accounted for the largest difference between the two cohorts, especially in the 5′-GpTpCp-3′ context (Fig. 2a). Next, we adopted a non-negative matrix factorization (NMF) algorithm^15^ to extract MSs from our exome sequencing data. Three prominent signatures were detected (Fig. 2b, c and Supplementary Fig.  2). Signatures MS2 and MS3 correspond to Catalog of Somatic Mutations in Cancer (COSMIC; https://cancer.sanger.ac.uk/cosmic/signatures/) Signature SBS5 and SBS40, respectively. SBS5 exhibits transcriptional strand bias for T > C substitutions in the ApTpN context, is found in most cancers and is correlated with age. In addition, the etiology of SBS40 is unknown, but the number of mutations attributed to SBS40 is correlated with patient age for some types of human cancer. Signature MS1, which corresponds to COSMIC Signature SBS22 and has been associated with exposure to AA, was only observed in the Chinese cohort (Fig. 2c and Supplementary Fig.  2). AA is a potential risk factor for several cancer types, including urothelial cell carcinoma^16,17^ and liver cancer^18^. In our cohort, we observed the AA signature in 26.3% of patients, indicating that there exists a special mutagenic process in Chinese ccRCC.

Previous reports suggest that ccRCC is a cancer with modest mutation load compared to other cancers^19^. However, in some of our patients, we observed a hyper-mutation phenotype. We divided our patients into two groups (AA and non-AA) based on whether the AA signature was obvious (weights >13%). The mutational burden of the AA group was higher than that of the non-AA group (median value: 101 vs. 52, Wilcoxon rank-sum test, *p* = 5.086e^−08^, Fig. 2d), and the mutation load increased with the AA weight (two-tailed *t* test, *p* = 5.072e^−12^, Fig. 2e). Moreover, our detection of the AA signature was cross-validated by mSigAct^18^ (Supplementary Fig.  3). It is worth noting that AA patients had significantly higher mutation frequencies in *CSMD3* (22.5% vs. 6.25%, Fisher’s exact test, *p* = 0.01273, Fig. 2f).

### Diverse mutation patterns between Chinese ccRCC and ccRCC-TT

**Fig. 3:**
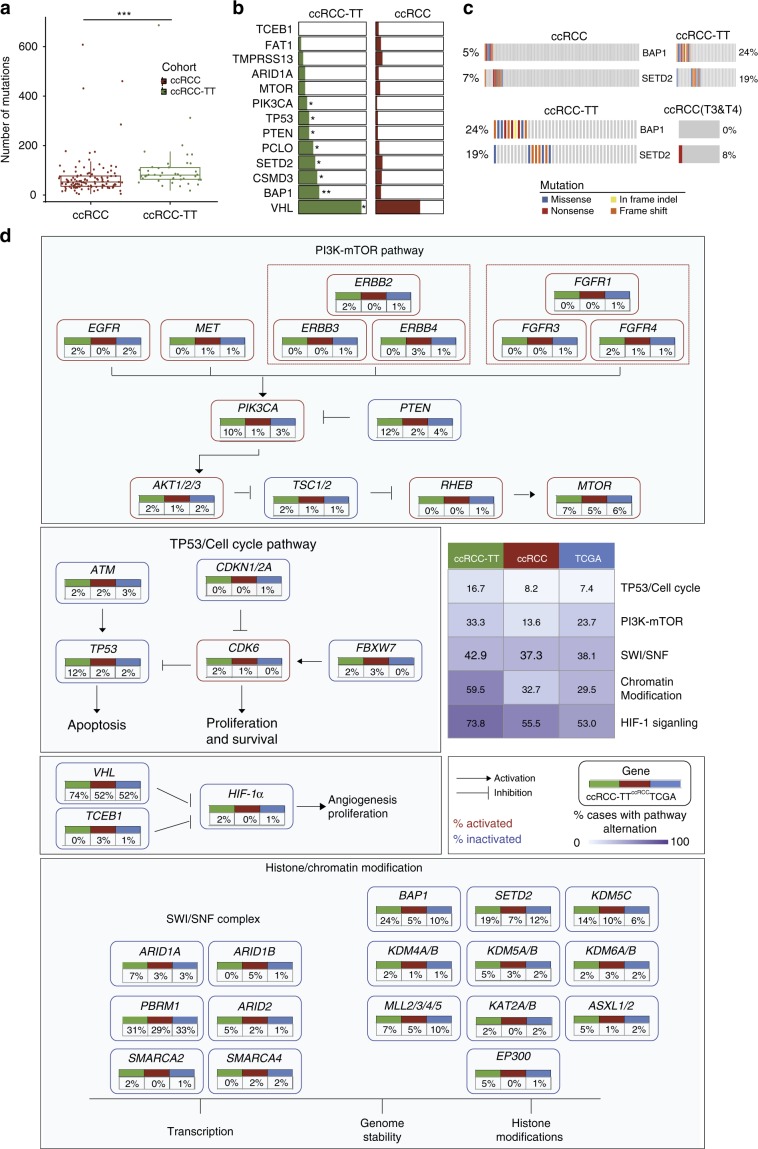
Diverse mutation patterns between Chinese ccRCC and ccRCC-TT cohorts. **a** The mutation load was compared between Chinese ccRCC and ccRCC-TT cohorts. The box plot displays the first and third quartiles (top and bottom of the boxes), the median (band inside the boxes), and the lowest and highest point within 1.5 times the interquartile range of the lower and higher quartile (whiskers). Wilcoxon rank-sum test, *p *<* 0.05, **p *<* 0.01, ***p *<* 0.001. **b** Nine significantly different mutated genes between the ccRCC and ccRCC-TT cohorts. Fisher’s exact test, *p *<* 0.05, **p *<* 0.01, ***p *<* 0.001. **c** Distribution of *BAP1* and *SETD2* mutations in ccRCC samples, ccRCC samples in the late stage and ccRCC-TT samples. *BAP1* and *SETD2* mutations were enriched in patients with TT. **d** Somatic mutations in signaling pathways across three cohorts. Non-silent mutations and indels were counted. The table shows the fraction of samples with alterations in each of the selected signaling pathways. In the pathway chart, the edges show pairwise molecular interactions, whereas boxes outlined in red denote alterations leading to pathway activation, whereas boxes outlined in blue indicate inactivation. The source data underlying Fig.  3a–c are provided as a Source Data file.

We investigated the genomic differences between two Chinese cohorts with the goal of revealing genomic features associated with the presence of a TT. We divided 152 patients into two cohorts according to whether the patient had a TT: a ccRCC cohort (*n* = 110) and a ccRCC-TT cohort (*n* = 42). In general, the primary tumors of ccRCC-TT patients showed a higher mutational burden compared with those of ccRCC patients (Median_ccRCC-TT_ = 80.5, Median_ccRCC_ = 52.5, Wilcoxon rank-sum test, *p* < 0.001, Fig. 3a). Moreover, we observed that *BAP1*, *CSMD3*, *TP53*, *SETD2*, *PTEN*, *PCLO*, *PIK3CA,* and *VHL* were mutated at a higher frequency in ccRCC-TT patients (Fig. 3b). Specifically, mutations in *BAP1*, an important gene that is involved in chromatin dynamics, is associated with a high risk for metastasis in uveal melanoma^20^ and is known as a tumor suppressor in ccRCC^21^, occurred more frequently in ccRCC-TT patients in comparison with ccRCC patients (24% vs. 5%, Fisher’s exact test, *p* = 0.002186, Fig. 3b). In addition, Chinese ccRCC-TT patients had a higher mutation frequency of *SETD2* (19% vs. 7%, Fisher’s exact test, *p* = 0.04278, Fig. 3b), a H3K36 methyltransferase whose inactivation promoted renal cancer branched evolution^22^ and whose overexpression in gastric cancer cell lines significantly inhibited cell proliferation, migration, and invasion^23^. To exclude the possibility that the difference was caused by tumor staging, we selectively analyzed late-stage ccRCC patients (stage > T3). Consistently, *BAP1* and/or *SETD2* mutations were highly enriched in ccRCC-TT patients (40% vs. 8%, Fisher’s exact test, *p* = 0.04379), which was in agreement with a previous study^24^ (Supplementary Fig.  4). Permutation tests showed that *BAP1* mutations were mutually exclusive with *SETD2* mutations (Fig. 3c). We also observed mutual exclusivity between *BAP1* mutation and *SETD2* mutation in the TCGA data, and survival analysis of *BAP1*/*SETD2* status showed different outcomes for the two types of events, with cases with *BAP1* or *SETD2* mutation exhibiting worse overall survival (OS) in comparison with wild-type individuals (median OS 31.2 vs. 37.9 months, *p* = 0.0016, log-rank test, Supplementary Fig.  5). In addition, DNA replication and base excision repair pathways were highly enriched in tumors with *BAP1* or *SETD2* mutations in comparison with tumors lacking these mutations (Supplementary Fig.  6). These findings suggested that *BAP1* and *SETD2* might be functionally redundant, which is consistent with knowledge regarding their roles in chromatin remodeling. These results highlighted that ccRCC patients with *BAP1* or *SETD2* mutations in the primary tumor are more prone to develop a thrombus.

In addition, we compared changes in typical cancer-related pathways between Chinese ccRCC and ccRCC-TT patients (Fig. 3d). Overall, ccRCC-TT had more alterations in all selected pathways/modules. In particular, the level of alterations in the chromatin modification pathway was significantly higher in ccRCC-TT in comparison with ccRCC (59.5% vs. 32.7%, Fisher’s exact test, *p* = 0.003186). Taken together, these results suggested that dysfunction of the chromatin remodeling pathway is critical for the occurrence of a thrombus in ccRCC patients.

### Genomic comparison between the thrombus and primary tumor

**Fig. 4:**
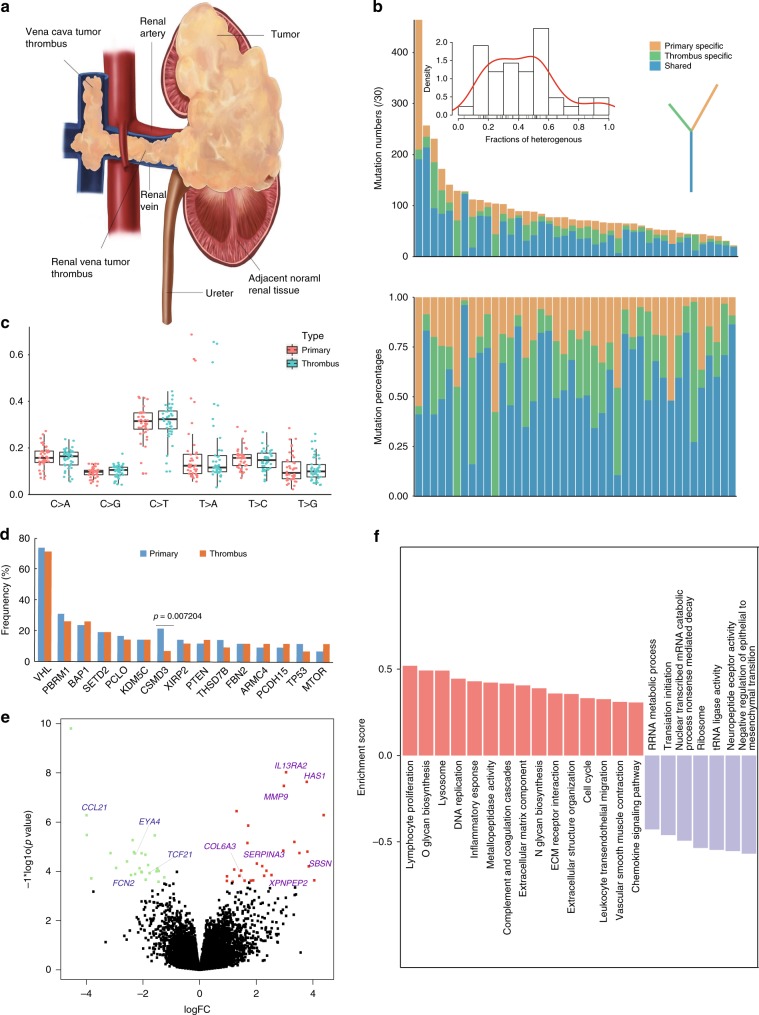
Genomic differences between primary tumors and thrombi. **a** Anatomical diagram of patients with thrombus. **b** The top bar plots display the numbers of shared or specific non-silent mutations between primary tumors and thrombi from 42 ccRCC-TT patients. The inset plot shows the distribution of heterogeneity between the primary tumors and thrombi. The bottom bar plots show the proportions of shared or specific mutations. **c** The contributions of six substitution patterns in primary tumors and thrombi. **d** The top 15 mutated genes in primary tumors and thrombi. The box plot displays the first and third quartiles (top and bottom of the boxes), the median (band inside the boxes), and the lowest and highest point within 1.5 times the interquartile range of the lower and higher quartile (whiskers). **e** Volcano plot of differentially expressed genes between primary tumors and thrombi with a threshold fold-change of 2 and *p* < 0.01. **f** The GSEA results revealed that the set of genes expressed in the thrombi were enriched in the cell cycle pathway, immunological pathway, and change of extracellular matrix and structure pathway. The source data underlying Fig.  4b–d are provided as a Source Data file.

Next, we explored the clonal relationship between primary tumors and thrombi in our ccRCC-TT cohort. We first analyzed the regional distribution of nonsynonymous mutations. Mutations were classified as shared mutations if they occurred in both the primary tumor and thrombus, and specific if they were only detected in either sample. The percentage of specific mutations in each ccRCC-TT case ranged from 3.9% to 100%, with an average of 43.44%, demonstrating a variable extent of genomic heterogeneity between the primary tumor and thrombus (Fig. 4b). In addition, the mutational spectra of the two cohorts were similar (Fig. 4c). Although some primary tumors and thrombi displayed ongoing evolution leading to specific mutations, most putative driver mutations, such as those in *VHL*, *BAP1,* and *SETD2*, were shared by two regions (Fig. 4d and Supplementary Fig.  7). Interestingly, there was no shared mutation between the primary tumor and thrombus in patients C032 and C042, which was indicative of multi-clonal origin. One possible explanation for this finding was that the thrombus stemmed from another clonally independent primary tumor.

Next, we compared the transcriptomes of primary ccRCC tumors and normal kidney tissue. We identified 2334 upregulated genes and 2176 downregulated genes in our tumor samples (Supplementary Fig.  8a). To investigate potentially altered pathways in the tumor samples, Gene Set Enrichment Analysis (GSEA)^25^ was implemented to compare the normal and tumor groups. We observed the activation of the immune response and many cancer-associated pathways, such as the cell cycle, mismatch repair, and TP53-signaling pathways (Supplementary Fig.  8b, c).

Comparison of the gene expression profiles of the primary tumors and thrombi revealed that 25 genes were significantly upregulated in the thrombi, including *MMP9*, *SBSN*, *XPNPEP2,* and *IL13RA2* (Fig. 4e). These genes were mostly associated with cell migration and invasion. *MMP9* is a member of the matrix metalloproteinase family, which has a role in degradation of the extracellular matrix and promotes tumor invasion and metastasis^26^. *XPNPEP2* (also known as aminopeptidase P) was reported to facilitate cervical cancer cell invasion and migration by mediating the EMT)^27^. GSEA^25^ showed that 15 pathways were significantly enriched in thrombi, most of which were related to the immune response, such as the lymphocyte proliferation, lysosome, and inflammatory response pathway. This finding might be explained by the venous microenvironment of the thrombus, which allows more immune cells to infiltrate the tumor (Fig. 4f).

### Characterization of gene expression subtypes in Chinese ccRCC

**Fig. 5:**
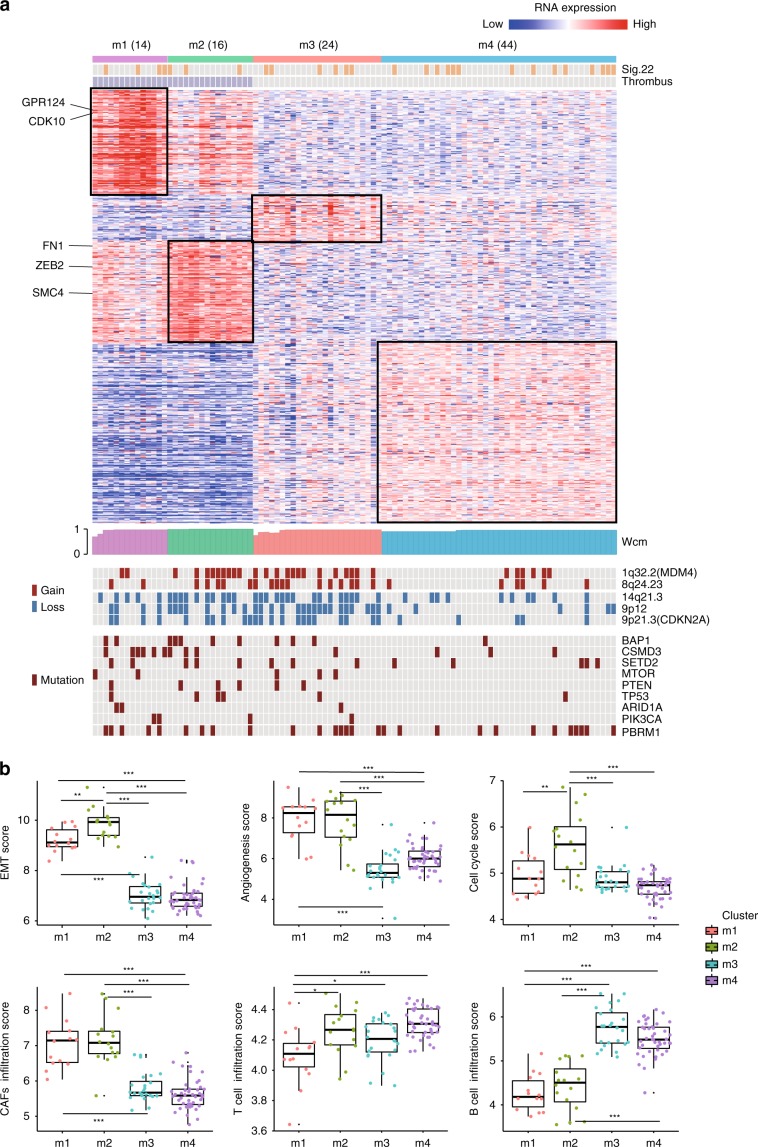
Gene expression subtypes. **a** Tumors were separated into four clusters by unsupervised analyses based on differentially expressed mRNA patterns (showing 540 representative genes). Top to bottom: AA signature, tumor stage information; normalized abundance heatmap for 98 mRNAs; profile of silhouette width calculated from the consensus membership heatmap, Wcm; covariates for recurrent copy number alteration regions, and mutations in *BAP1*, *CSMD3*, *SETD2, MTOR, PTEN, TP53, ARID1A*, *PIK3CA,* and *PBRM1*. Some important genes related to the cell cycle, angiogenesis, and the EMT are listed on the left. **b** Overall, the scores of gene sets associated with EMT, angiogenesis, and cell cycle process in patients with thrombus were increased compared with patients with no thrombus. The phenomenon of immune cell infiltration was complex; CAF infiltration was more obvious in patients with thrombus, but T-cell and B-cell infiltration was more common in non-thrombus patients. Each dot presents one sample. The box plot displays the first and third quartiles (top and bottom of the boxes), the median (band inside the boxes), and the lowest and highest point within 1.5 times the interquartile range of the lower and higher quartile (whiskers). Wilcoxon rank-sum test, *p *<* 0.05, **p *<* 0.01, ***p *<* 0.001. The source data underlying Fig.  5a are provided as a Source Data file.

Using an unsupervised clustering method, we identified four gene expression clusters in the Chinese cohort. Interestingly, samples were clustered by TT status instead of tumor stage or AA signature, suggesting that patients with TT had distinct transcriptomic profiles (Fig. 5a). Clusters m1 and m2 were two TT subtypes. The m1 cluster showed upregulation of the autophagy pathway and higher frequencies of *CSMD3* mutations (42.9% in m1 vs. 8.3% in others, Fisher’s exact test, *p* = 0.003); this cluster also harbored a greater number of *ARID1A* mutations (14.3% vs. 1.2%, Fisher’s exact test, *p* = 0.053) and *PIK3CA* mutations (14.3% vs. 2.4%, Fisher’s exact test, *p* = 0.097). Some genes associated with DNA repair were upregulated in cluster m2, and *BAP1* mutations were more frequent in this cluster (31.3% vs. 7.3%, Fisher’s exact test, *p* = 0.016); however, this group also harbored more *SETD2* mutations (19% vs 12%, Fisher’s exact test, *p* = 0.24). Clusters m1 and m2 were both characterized by gene sets associated with angiogenesis and the epithelial–mesenchymal transition (EMT) process (Fig. 5b). Deletion of *CDKN2A* (50% vs 20.3%, Fisher’s exact test, *p* = 0.008) and amplification of *MDM4* (54.1% vs. 23.0%, Fisher’s exact test, *p* = 0.009) were more frequent in Cluster m3 (Fig. 5a). We also applied supervised clustering to investigate the similarities and differences between our mRNA subtypes and the TCGA expression subtypes^3^. We found that samples in the two TT clusters (cluster m1 and m2) were separated from all cases without TT (Supplementary Fig.  9). Significant concordance was observed between our cluster m3 and the TCGA T3 subtype, and both two subtypes were characterized by a higher frequency of *CDKN2A* deletion (Fig. 5a and Supplementary Table 2). Our Cluster m4 showed similarity to the TCGA T1 subtype, but *PBRM1* mutations, which are common in the TCGA T1 subtypes were not enriched in cluster m4 (Supplementary Table 2).

Next, based on cell-type-specific expression markers, we inferred the composition of the tumor microenvironment from transcriptome data^28^. We found that clusters m1 and m2 exhibited higher abundance of CAFs but fewer T cells and B cells (Fig. 5b). We also performed CIBERSORT^29^ analysis on our data, which revealed that there were fewer T cells and B cells in the tumor microenvironments of clusters m1 and m2 (Supplementary Fig.  10).

## Discussion

In this work, we present an integrative genomic study of Chinese ccRCC and reveal features specific to the Chinese population. Compared with the TCGA cohort, we found a higher prevalence of *CSMD3* (11%) and *TMPRSS13* (7%) mutations in Chinese patients. We also observed more copy number (CN) aberrations in the Chinese cohort.

AA is a natural product of plants of the genus *Aristolochia*, which are widely used in herbal remedies and health supplements. As a class 1 carcinogen, AA can bind DNA and form DNA adducts that have been implicated in carcinogenesis of urothelial cell carcinoma^16,17,30^ and liver cancer^18^. Notably, we found that the AA signature was detected in 26.3% of patients in the Chinese ccRCC cohort, but it was not detected in any patient in the TCGA cohort. We also observed a positive correlation between the AA signature and the mutational load of Chinese ccRCC. In particular, patients with a hypermutator phenotype were mainly characterized by the AA signature (Supplementary Fig.  11).

Intravascular tumor growth is a feature of ccRCC, and TT formation generally indicates a poor prognosis^8^. In the current study, we found that patients with TT harbored significantly higher frequencies of mutations in *BAP1* or *SETD2*. Interestingly, *BAP1* and *SETD2* mutations were mutually exclusive in our cohort, possibly because these two genes are functionally similar; both genes are involved in the chromatin remodeling pathway. In a recent study, Turajlic et al.^24^ described a multiple clonal drivers-subtype that sometimes involves *BAP1* and *SETD2* mutations in the same patient, but they also claimed that they generally observed mutual exclusivity between *BAP1* and *SETD2* mutations at the clonal level. Moreover, *BAP1* and *SETD2* mutations were uniformly observed as shared mutations in both primary tumors and thrombi, indicating that mutations in these two genes occur relatively early during tumorigenesis. This finding suggests that the thrombosis process is a predetermined event that may be associated with *BAP1* or *SETD2* mutations in primary tumors. Previous studies reported that mutations in *BAP1* or *SETD2* were associated with worse survival for ccRCC patients^31^. However, the mechanism underlying this effect was unclear. Our results reveal that mutations in *BAP1* or *SETD2* were highly enriched in patients with TT, which may indicate that the poor prognosis associated with *BAP1* or *SETD2* mutations is owing to the formation of a TT. Meanwhile, tumors from ccRCC-TT patients represent distinct molecular subtypes and microenvironment compositions, which may have implications for ccRCC diagnosis, prognosis, and treatment.

## Methods

### Sample selection

This study was approved by local ethics committees (Peking University Third Hospital and Chinese PLA General Hospital), and written informed consent was obtained from all patients. Patients were included if they had histologically confirmed ccRCC and received no treatment before surgery. Tumors and matched blood or normal tissues were obtained from 152 nephrectomy patients. Sample collection was performed according to strict standard operating procedures in all cases and was documented by photography. The specimens were collected immediately following nephrectomy and flash frozen in liquid nitrogen. Patient characteristics and clinical information are shown in Supplementary Table 1 and Supplementary Data 1, but the survival data associated with our patient cohort are currently unavailable. Sequencing depth and coverage information is summarized in Supplementary Data 2.

### Library construction and sequencing

DNA was extracted using the Qiagen AllPrep kit following the manufacturer’s instructions. DNA degradation and suspected RNA/protein contamination were verified by electrophoresis on 1% agarose gels. The concentration and purity of DNA samples were quantified precisely by the Qubit dsDNA hs assay kit in a Qubit3.0 Fluorometer (Life Technologies, CA, USA). A total amount of 0.4 μg DNA per sample was required for library preparation for exome sequencing. RNA degradation and contamination were monitored on 1% agarose gels. The exome sequences were enriched from 0.4 μg genomic DNA using NimbleGen’s SeqCap EZ and Agilent liquid capture system (Agilent SureSelect Human All Exon V6) according to the manufacturer’s protocol. The libraries were sequenced on an Illumina Hiseq X Ten platform and 150 bp paired-end reads were generated.

RNA from 30 TT paired samples was extracted using the Qiagen AllPrep kit following the manufacturer’s instructions. RNA from 61 normal-tumor paired samples and 7 tumor samples was isolated using the Ribo-ZeroTM Gold Kit. RNA purity was checked using a NanoPhotometer spectrophotometer (IMPLEN, CA, USA). RNA concentrations were measured using the Qubit® RNA Assay Kit in a Qubit2.0 Fluorometer (Life Technologies, CA, USA). RNA integrity was assessed using the RNA Nano 6000 Assay Kit with a Bioanalyzer 2100 system (Agilent Technologies, CA, USA). A total amount of 3 µg RNA per sample was used as the input material for RNA sample preparation. Sequencing libraries were generated using the NEBNext UltraTM RNA Library Prep Kit for Illumina (NEB, USA) following the manufacturer’s recommendations, and index codes were added to the attribute sequences of each sample. Clustering of the index-coded samples was performed on a cBot Cluster Generation System using the TruSeq PE Cluster Kit v3-cBot-HS (Illumina) according to the manufacturer’s instructions. After cluster generation, the library preparations were sequenced on an Illumina Hiseq X Ten platform, and 150 bp paired-end reads were generated.

### Processing of exome sequencing data

Paired-end read sequences were aligned to human genome hg19 (UCSC) using the Burrows-Wheeler Aligner (BWA) with default parameters^32^, after which they were sorted and merged by SAMtools 0.1.19 (ref.^33^). Picard (v1.76) was applied to fix mate pairs and mark and discard duplicates (http://Picard.Sourceforge.net). Next, realignment of all insertions and deletions (INDELs) and base quality recalibration were carried out using the Genome Analysis Toolkit (GATK 2.1–8)^34^.

Somatic substitutions (SNV) were called using the MuTect module in GATK with normal kidney tissue or blood samples from the same patient as the control group. InDels were detected by the GATK Unified Genotyper. In order to accurately detect reliable SNVs and InDels, we used a set of strict filtration criteria: (1) the number of reads covering the mutated sites should not be fewer than 10, with at least three reads harboring the mutations; (2) at least 10× coverage for normal samples was required, with at most one read harboring the mutations; (3) the minimum value of the maximum mapping quality score for mutated alleles was set to 20; (4) the mutation allele frequency was at least 8%; (5) mutations listed in dbSNP 135 were removed unless they were documented by the Catalog of Somatic Mutations in Cancer (COSMIC); (6) mutations reported by the National Heart, Lung, and Blood Institute Exome Sequencing Project were filtered out. All InDels were manually checked to ensure fidelity. Snpeff 3.0 (ref.^35^) was used to annotate all SNVs and InDels. To reduce the risk of false negative calls, we also lowered the requirement of mutation allele frequency to 5% and coverage to at least 5×, similar to the criteria used in the TCGA study^3^, which generated similar mutation calling results.

### CN analysis

To portray CN states across the whole genome based on WES data, Sequenza R v.2.1.1 (ref.^36^) was applied to model CNs to integers with consideration of both ploidy and cellularity. Standard BAM files of samples with their matched normal controls were used as input to calculate the depth ratio and normalized ratio with consideration of both GC content and data quality. To estimate purity and ploidy, the following parameters were used: breaks.method = full, gamma = 40, kmin = 5, gamma.pcf = 200, and kmin.pcf = 200. In addition, the processed segmented CN data from Sequenza were used as input for GISTIC2 (ref.^14^) to identify significantly amplified/deleted regions with the default parameters. A default *q* value threshold (0.25) was used to define highly amplified/deleted regions.

### MS analysis

We investigated the mutational spectrum of 96 subtypes of three-base context of mutations, considering six substitution patterns (C > A, C > G, C > T, T > A, T > C, and T > G) and 5′- and 3′-flanking nucleotides for all WES mutation data. To extract the underlying MSs from the mutational spectrum, we adopted a NMF-based method, sigProfiler^15^. Moreover, we used the cosine similarity distance to measure the similarity between our identified signatures and COSMIC v3 MSs (https://cancer.sanger.ac.uk/cosmic/signatures). The siganazyler^37^ method was used to cross-validate our results with three deciphered MSs. To accurately assess whether the observed AA MS was present in each sample, we carried out a signature presence test using mSigAct^18^. The patient was considered to have the AA signature if both the mSigAct presence test and NMF method indicated AA exposure (Supplementary Fig.  7). The same analysis was applied to mutations called from the TCGA WES data set.

### Identification of driver mutations and comparison analysis

MutSigCV^10^ was used to identify SMGs. MutSigCV considers the overall mutation situation of the genome, the mutation frequency of genes near the mutation site, whether the site is located in a region where the chromosome is easy to open, and other parameters to find genes with a mutation rate higher than the calculated background mutation rate. Multiple testing correction (Benjamin–Hochberg false discovery rate) was performed, and genes with a *q* value < 0.05 were reported. Furthermore, we defined potential driver mutations if one of the following conditions was met: (1) mutations were documented by the COSMIC database (ccRCC-associated or related to another type of cancer; (2) mutations in the gene were identified by recent large-cohort ccRCC sequencing studies; (3) mutations in the gene were present in the KEGG (Kyoto Encyclopedia of Genes and Genomes) cancer pathways. Somatic mutations from the TCGA WES data of 417 ccRCC patients and clinical information were downloaded from the TCGA Data Portal (http://tcga-data.nci.nih.gov/docs/publincations/kirc_2013/) to allow us to compare the mutation rates of ccRCC genes. In addition, we evaluated somatic mutations at the gene level within the context of five well-studied ccRCC-associated signaling pathways: the TP53/cell cycle pathway, PI3K-mTOR pathway, SWI/SNF pathway, histone/chromatin modification pathway, and HIF−1 signaling pathway.

### RNA sequencing to assess gene expression

RNA reads were aligned to the hg19 genome assembly using HISAT2 (ref.^38^) and quantified with HTseq^39^. Cuffquant and cuffnorm^40^ were used to quantify the gene expression abundance and calculate the standardized gene expression (represented as FPKM) of each sample. The raw read counts were normalized with DESeq2 (ref.^41^) to estimate gene expression levels and identify differential gene expression. Differential gene expression was identified using a *p* value threshold of <0.05 and a fold-change threshold of at least 2.

### Unsupervised mRNA expression clustering

For unsupervised clustering analysis, the gene expression data for *N* = 98 samples were pre-processed to determine the most highly expressed and variable 1500 genes across the samples. We removed genes with NA values of >70% of all samples and then selected the top 1500 genes with the greatest variation by calculating the maximum absolute deviation of gene expression across the samples. The data were transformed into a non-negative matrix and clustered using non-negative matrix factorization^42^. The number of subtypes was selected by cophenetic coefficient and consensus clustering matrices. The gene normalization and selection methods used for supervised clustering were the same as those used in the TCGA study^3^. Scaled data were used as the input for a principal component analysis based on variable genes. The concordance of the derived expression subtypes was examined in comparison with subtypes published in the TCGA paper^3^ by the chi-squared test. The gene transcription signature score for the cell cycle was defined by the average relative expression of cell cycle-associated gene sets. The same metric was applied for gene sets associated with angiogenesis, EMT, cancer-associated fibroblast (CAF) infiltration, T-cell infiltration, and B-cell infiltration^28^.

### Survival analysis

To examine the correlations between *BAP1* and *SETD2* mutations and survival for the TCGA data^3^, 417 patient samples were separated into gene-mutated and wild-type subsets. Patient death was the endpoint, with follow-up time defined using the months_to_last_followup field if the patient was alive and the months_to_death field if the patient was deceased. Kaplan–Meier analysis was performed to compare survival for specific genes status, and the log-rank test was used to evaluate significant differences.

### GSEA

GSEA was performed to determine whether an a priori defined set of genes showed statistically significant, consistent differences between two biological states. The clusterProfiler^43^ was applied to the gene expression data to perform GSEA based on MSigDB (www.broadinstitute.org/gsea/msigdb) collections C2 and C5.

### Statistical analysis

All statistical analysis was conducted using R v3.5.3 (Foundation for Statistical Computing). The Wilcoxon rank-sum test and Fisher exact test were used to analyze genomic differences between the Chinese ccRCC and TCGA ccRCC cohorts, as well as between the Chinese ccRCC and Chinese ccRCC-TT cohorts, with regard to mutation rates and the fraction of the genome affected by CNAs. Unsupervised clustering was performed to identify gene expression subtypes.

### Reporting summary

Further information on research design is available in the Nature Research Reporting Summary linked to this article.

## Supplementary information

Supplementary Information

Description of Additional Supplementary Files

Supplementary Data 1

Supplementary Data 2

Reporting Summary

## Data Availability

The whole-exome and transcriptome-sequencing data have been deposited in the database of NCBI Sequence Read Archive (SRA) under accession code PRJNA596359 and PRJNA596338. The source data underlying Figs. 1a–c, 2c–f, 3a–c, 4b–d, and 5a, as well as Supplementary Figs. 4 and 10 are provided as a Source Data file.

## Source data

Source Data
