# Supplementary material for: Integrative genomic study of Chinese clear cell renal cell carcinoma reveals features associated with thrombus
Source: Nat Commun. 2020 Feb 6;11:739. doi: 10.1038/s41467-020-14601-9 (PMC7005298; doi:10.1038/s41467-020-14601-9)
Supplement: Supplementary file 1 — Supplementary Information [file 41467_2020_14601_MOESM1_ESM.pdf]

Supplementary Information for

Integrative Genomic study of Chinese clear cell renal cell carcinoma reveals features associated with thrombus

by Wang et al.

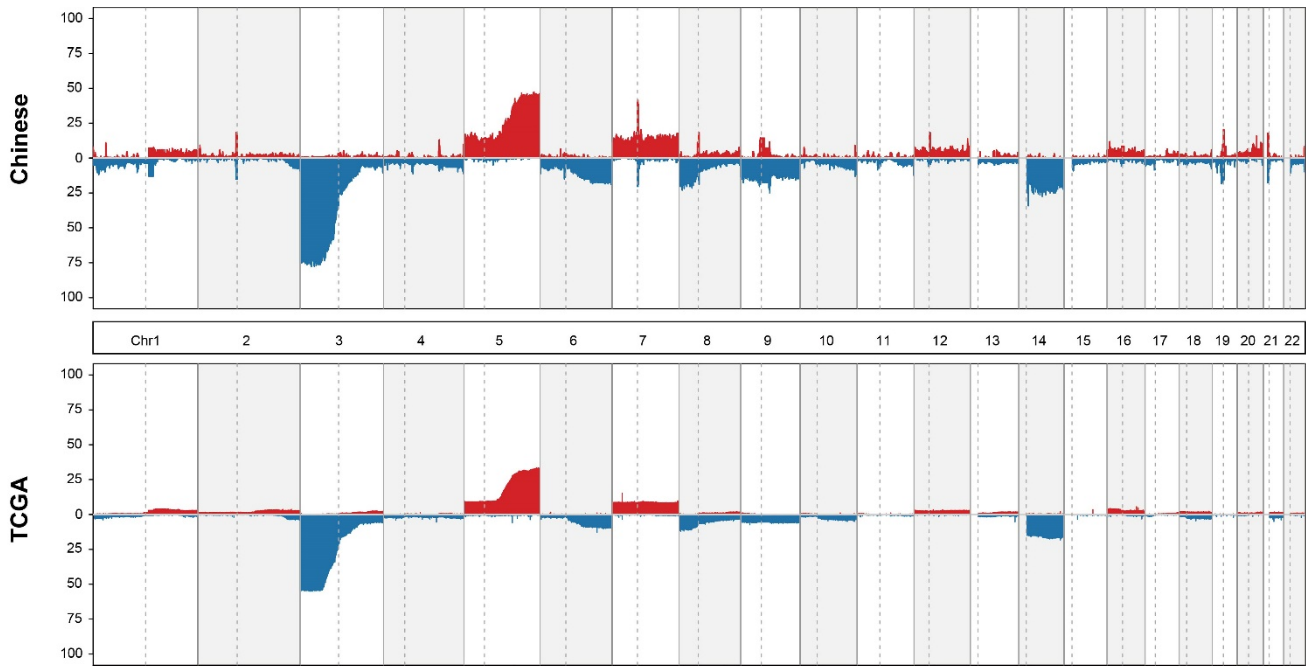

Supplementary Figure 1: Genome-wide copy number plots depicting chromosomal gains (red) and losses (blue) in the Chinese and TCGA samples.

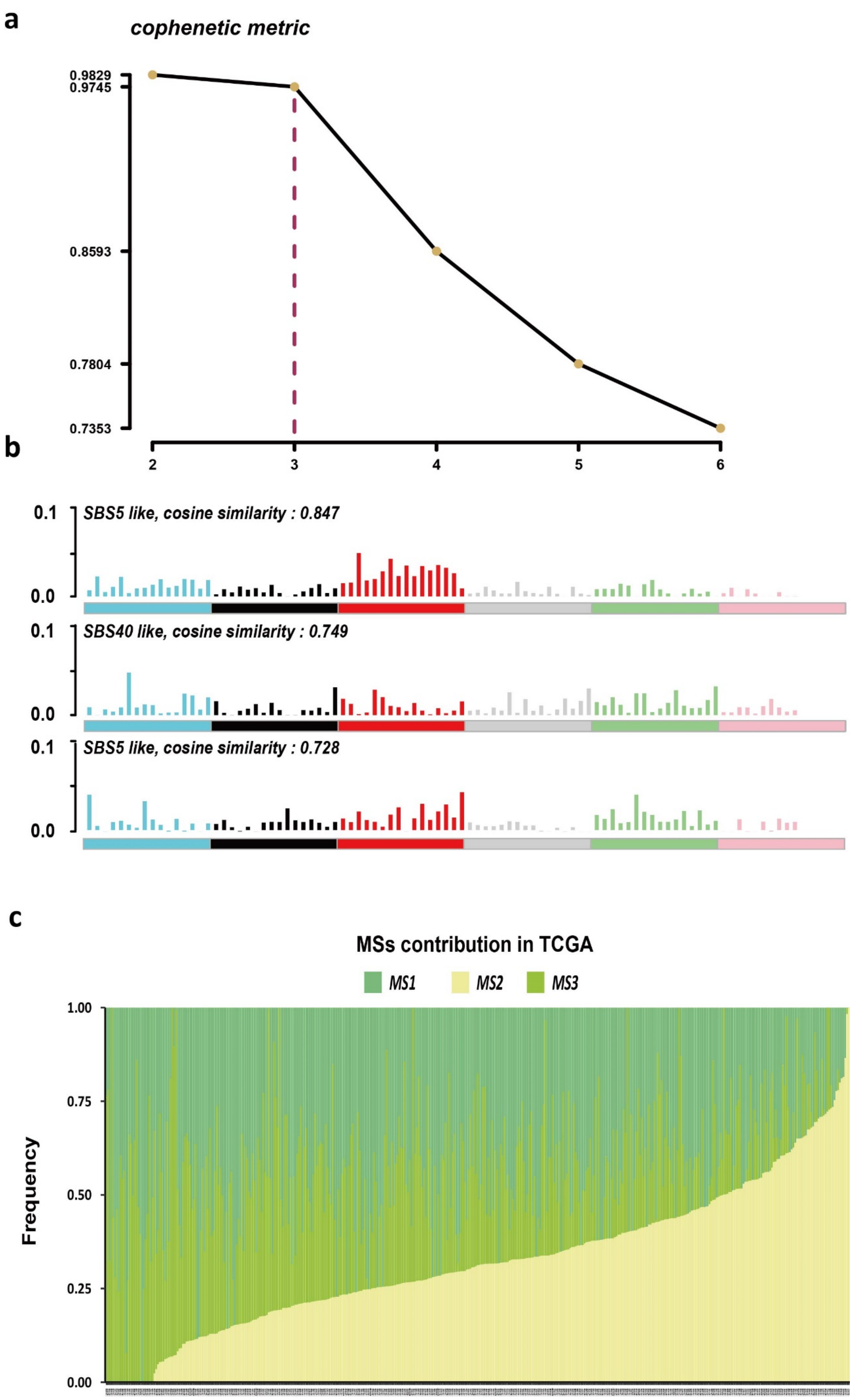
a
cophenetic metric
0.9829
0.9745
0.8593
0.7804
0.7353
2
3
4
5
6
b
0.1
0.0
SBS5 like, cosine similarity : 0.847
SBS40 like, cosine similarity : 0.749
SBS5 like, cosine similarity : 0.728
c
MSs contribution in TCGA
MS1
MS2
MS3
1.00
0.75
0.50
0.25
0.00
Frequency

Supplementary Figure 2: Mutational signatures and their activities in TCGA samples. a, The cophenetic metric plot showed that 3 signatures were deciphered. **b,** Mutational signature barplots. MS1 and MS3 correspond to SBS5, while MS2 corresponds to SBS40. These corresponding signatures are defined by COSMIC mutational signatures v3 (https://cancer.sanger.ac.uk/cosmic/signatures). **c,** Contributions of each mutational signature per sample.

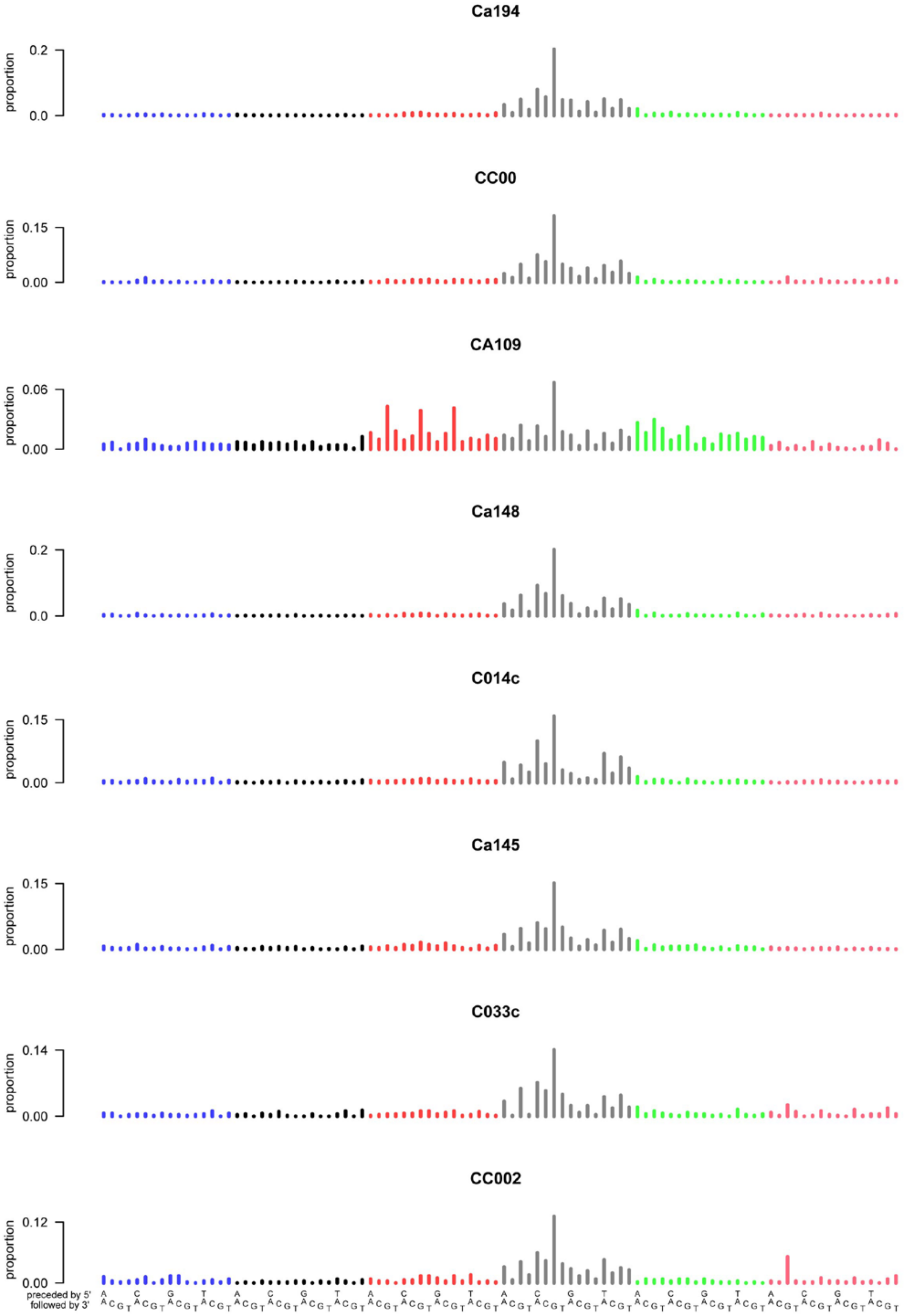
Ca194
proportion
0.2
0.0
CC00
proportion
0.15
0.00
CA109
proportion
0.06
0.00
Ca148
proportion
0.2
0.0
C014c
proportion
0.15
0.00
Ca145
proportion
0.15
0.00
C033c
proportion
0.14
0.00
CC002
proportion
0.12
0.00
preceded by 5'
followed by 3'
A C G T A C G T A C G T A C G T A C G T A C G T
ACGT ACGT ACGT ACGT ACGT ACGT ACGT ACGT ACGT ACGT ACGT ACGT ACGT ACGT ACGT ACGT ACGT ACGT ACGT ACGT ACGT ACGT ACGT ACGT

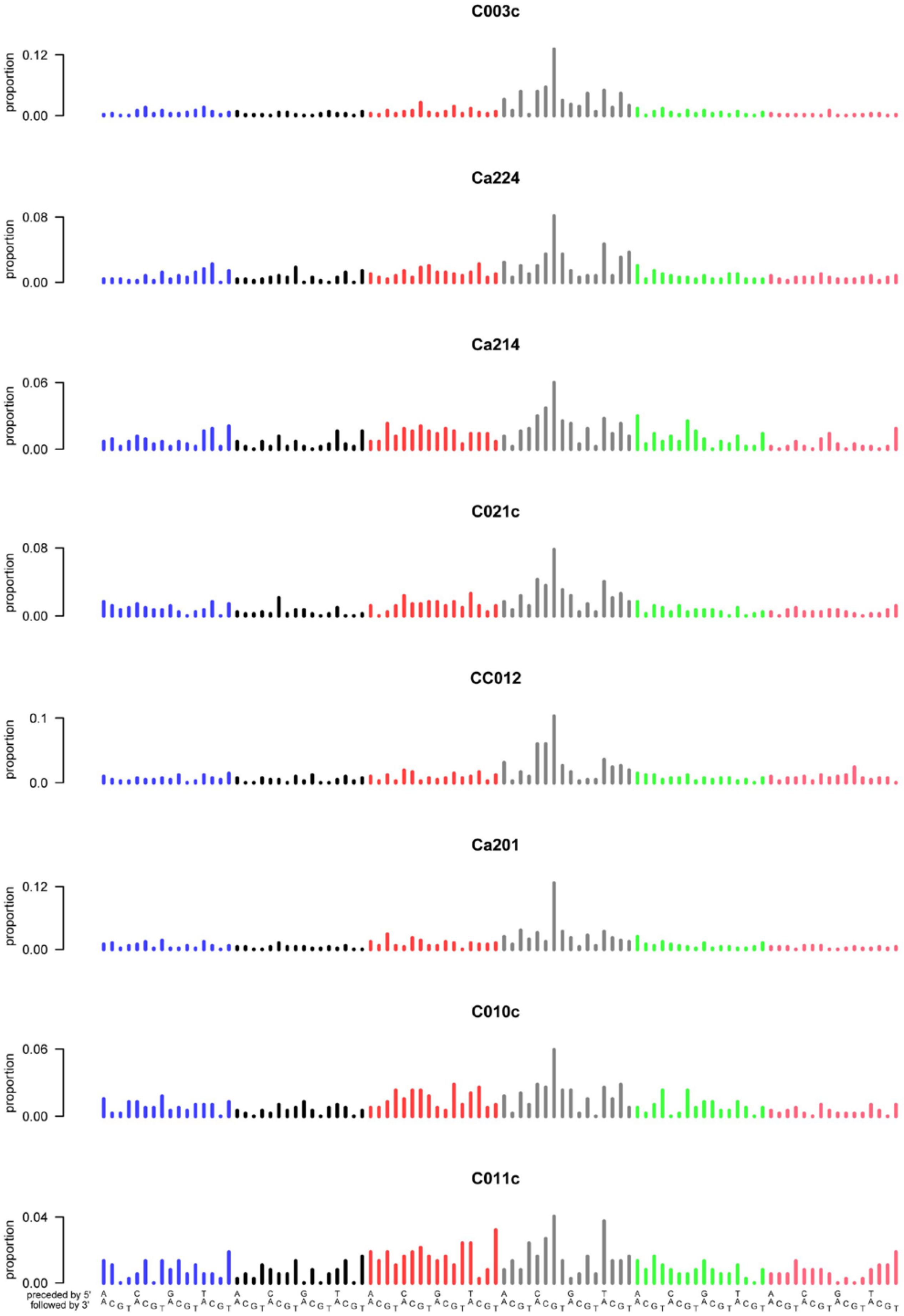
C003c
proportion
0.12
0.00
Ca224
proportion
0.08
0.00
Ca214
proportion
0.06
0.00
C021c
proportion
0.08
0.00
CC012
proportion
0.1
0.0
Ca201
proportion
0.12
0.00
C010c
proportion
0.06
0.00
C011c
proportion
0.04
0.00
preceded by 5'
followed by 3'
A C G T A C G T A C G T A C G T A C G T A C G T
ACGT ACGT ACGT ACGT ACGT ACGT ACGT ACGT ACGT ACGT ACGT ACGT ACGT ACGT ACGT ACGT ACGT ACGT ACGT ACGT ACGT ACGT ACGT ACGT

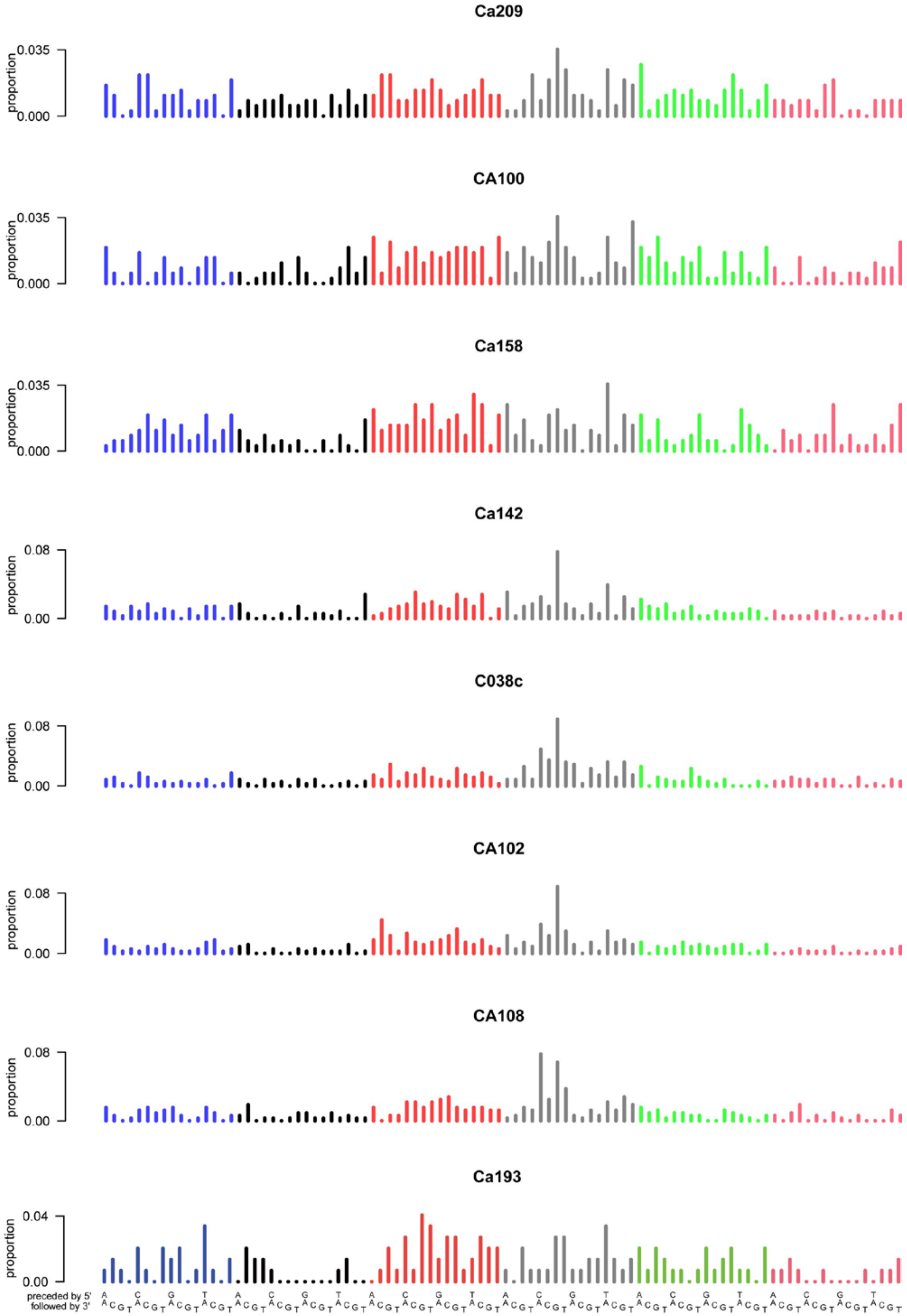

Ca209
CA100
Ca158
Ca142
C038c
CA102
CA108
Ca193
proportion
0.035
0.000
0.08
0.00
0.04
preceded by 5'
followed by 3'

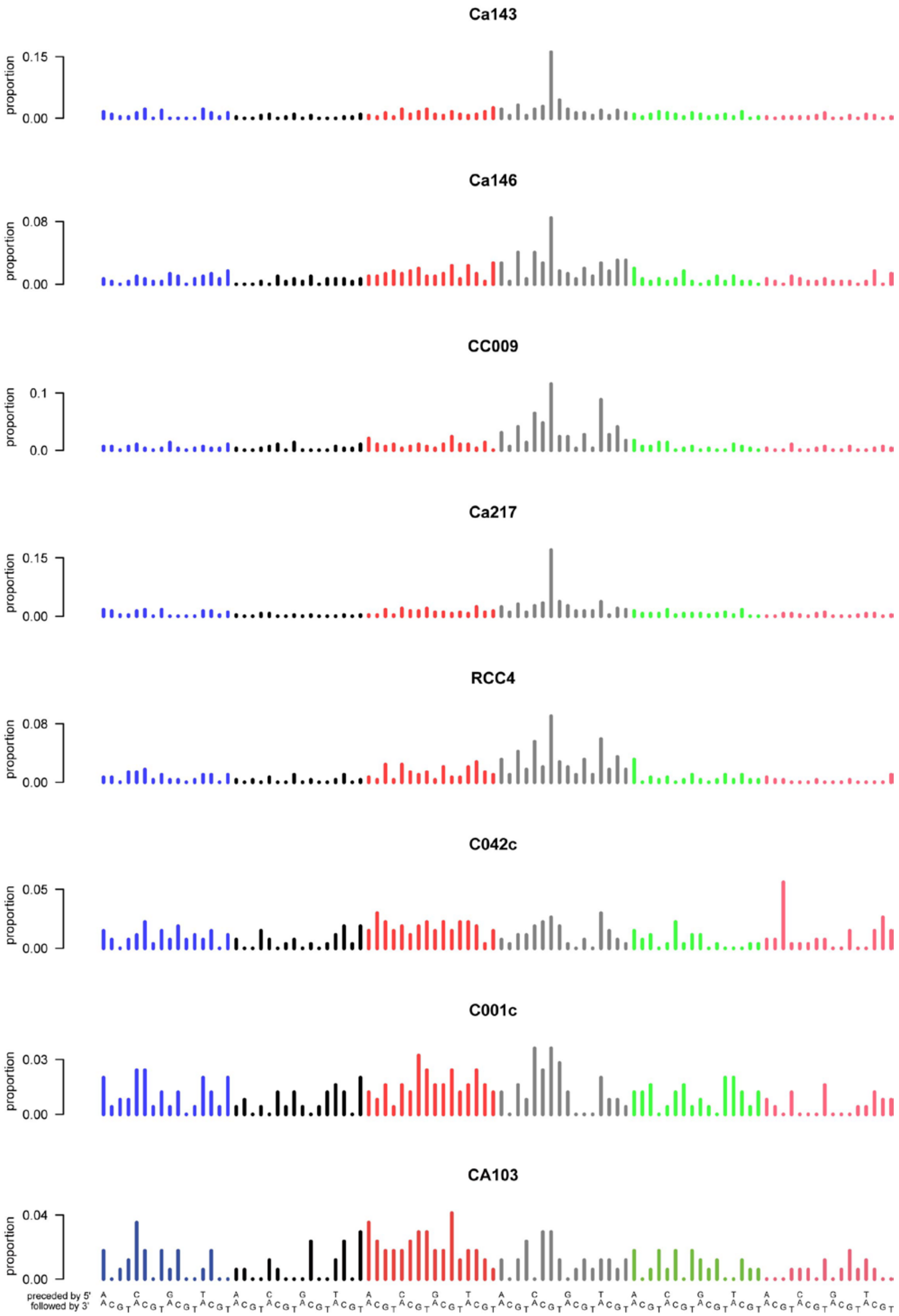
Ca143
proportion
0.15
0.00
Ca146
0.08
0.00
CC009
0.1
0.0
Ca217
0.15
0.00
RCC4
0.08
0.00
C042c
0.05
0.00
C001c
0.03
0.00
CA103
0.04
0.00
preceded by 5'
followed by 3'

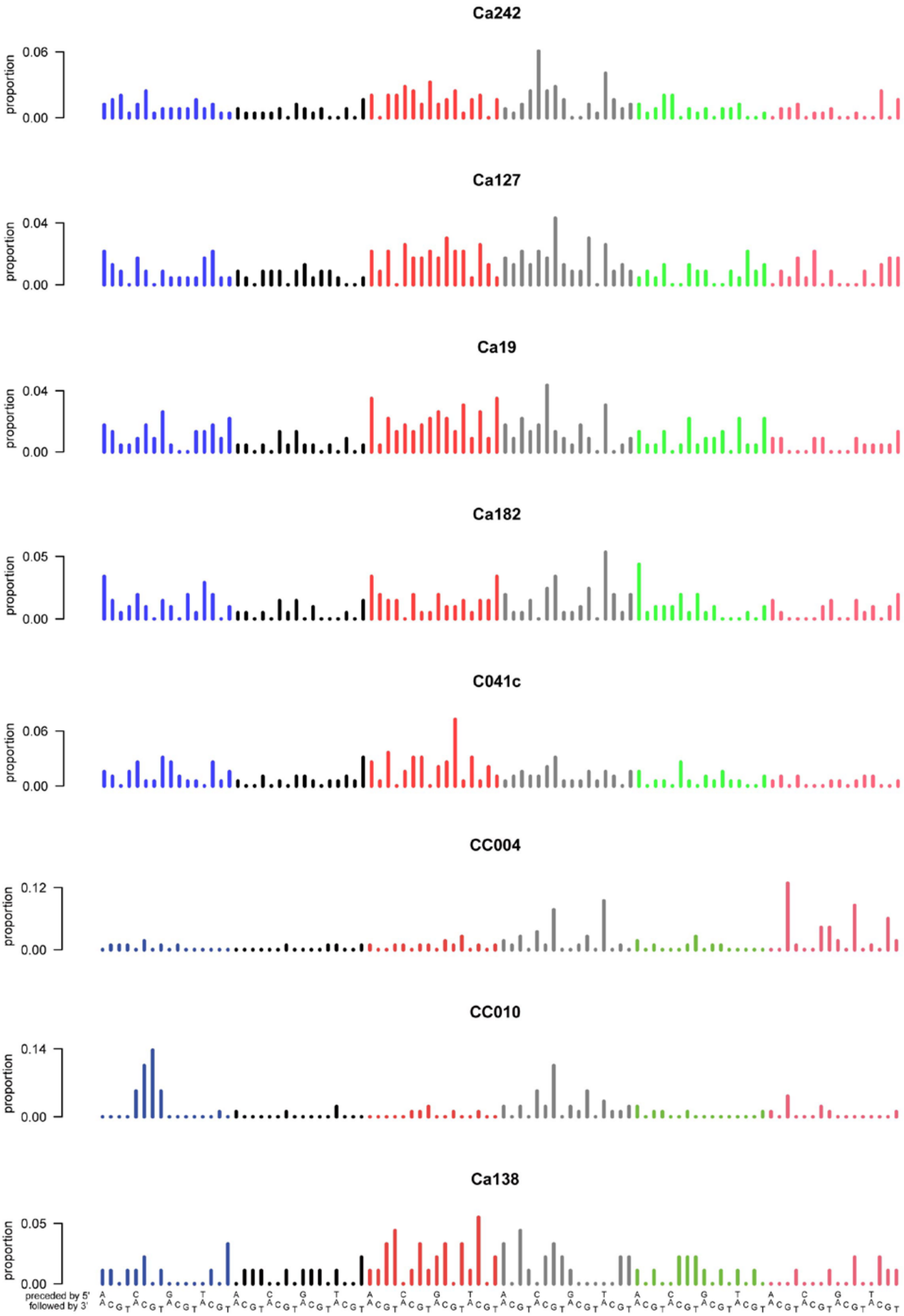

Supplementary Figure 3: Mutational spectra of 40 defined AA-dominant patients.

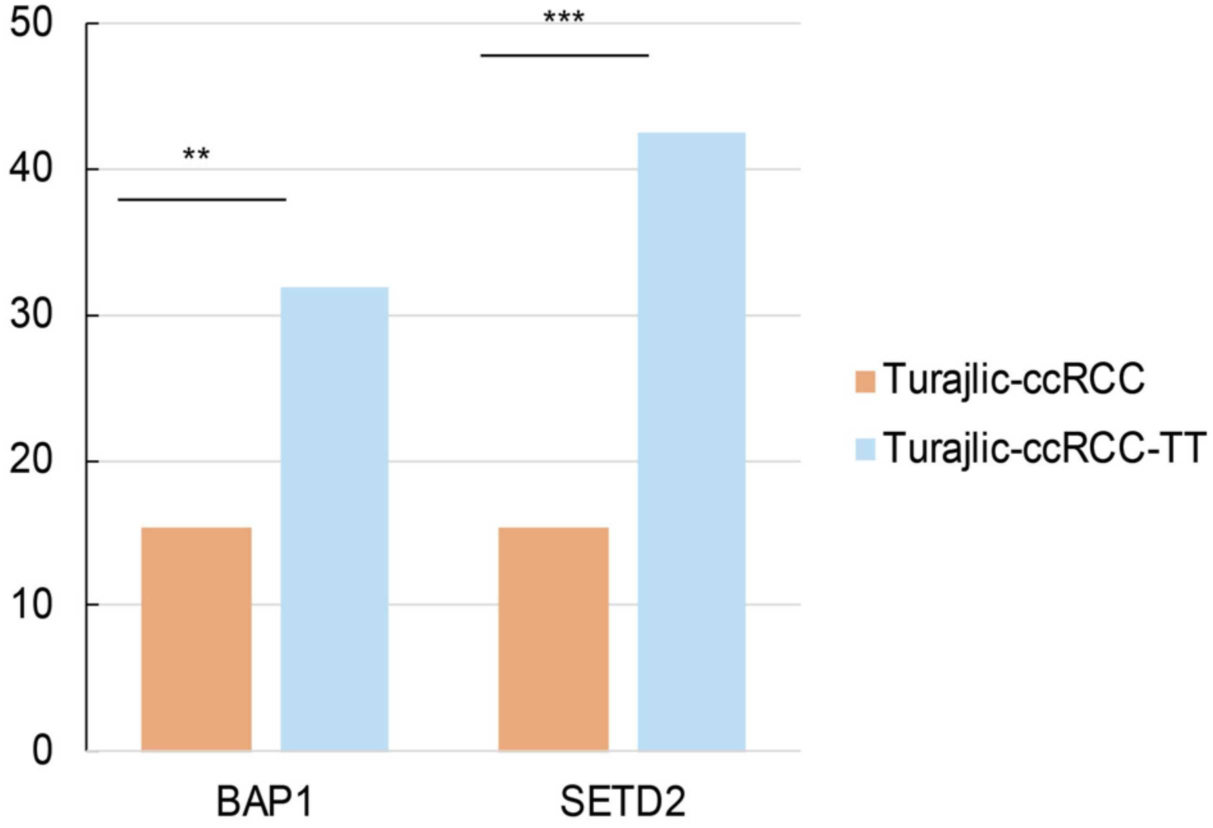

Supplementary Figure 4: The mutational frequencies of *BAP1* and *SETD2* in the late stage ccRCC cohort and ccRCC-TT cohort from Turajlic et al.'s study. Fisher's exact test, $*p < 0.05$, $**p < 0.01$, $***p < 0.001$. The source data underlying Supplementary Fig. 4 is provided as a Source Data file.

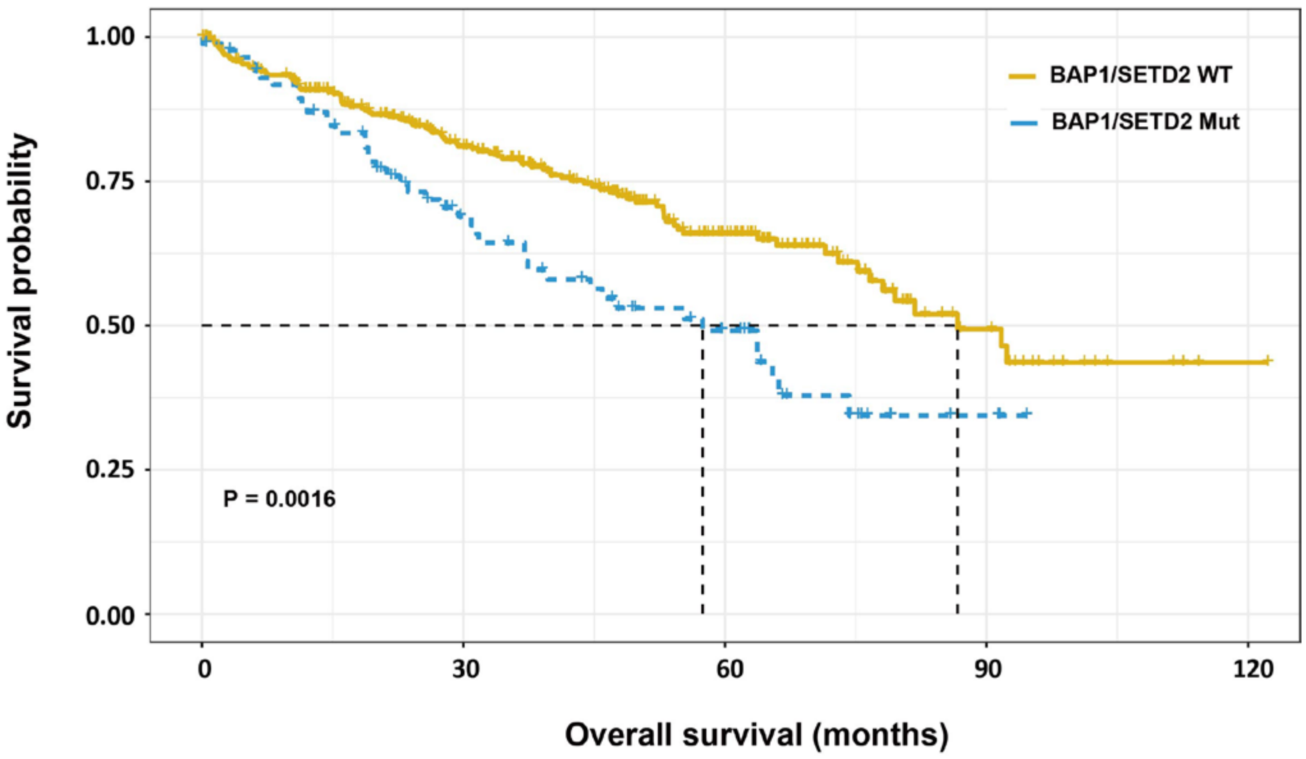

Supplementary Figure 5: Survival analysis for TCGA cohort. Significant difference in patient survival was identified for *BAP1* and/or *SETD2* mutations.

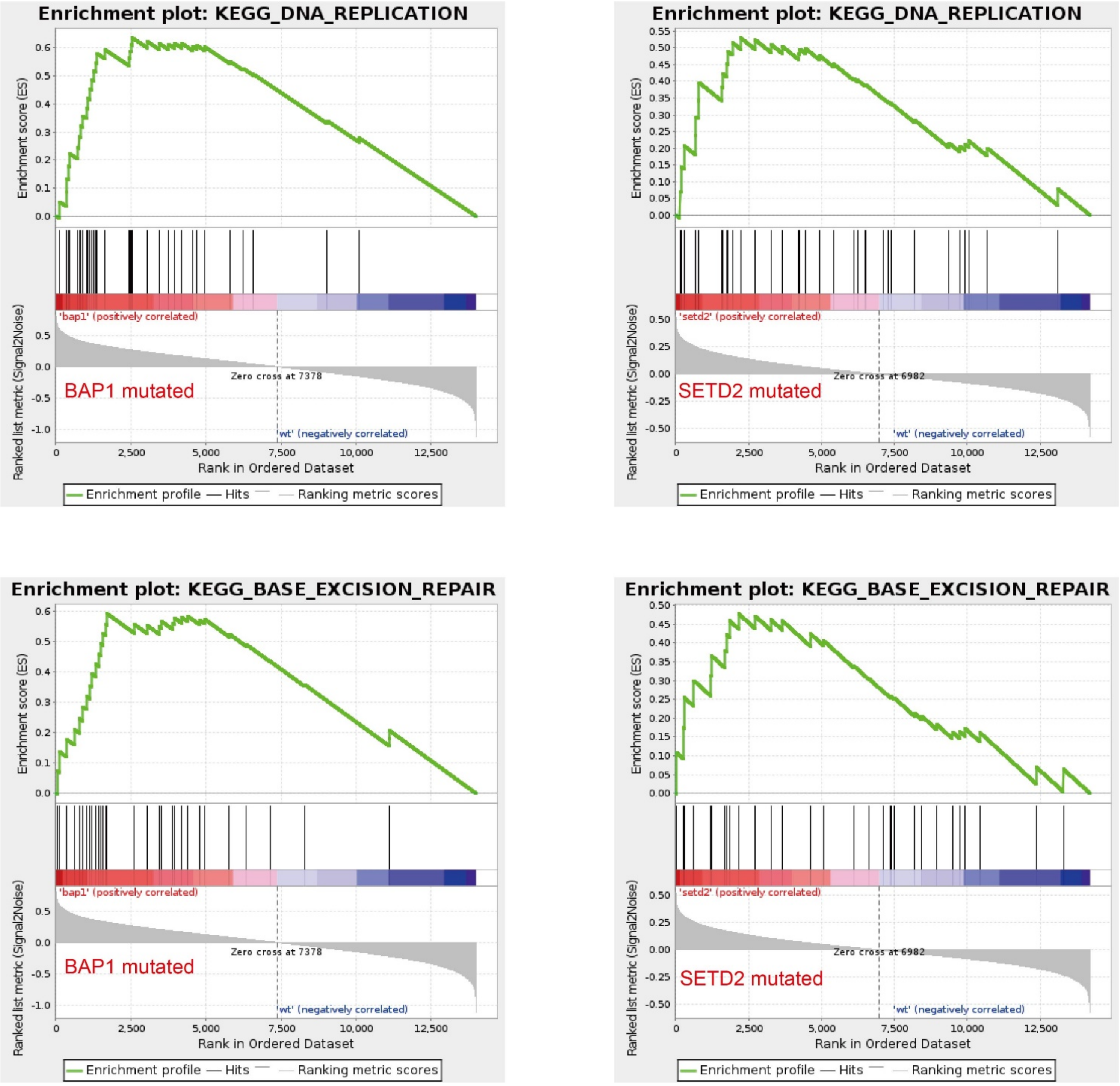

Supplementary Figure 6: Enrichment plots for tumors with BAP1 or SETD2 mutations compared with BAP1 and SETD2 wild-type tumors.

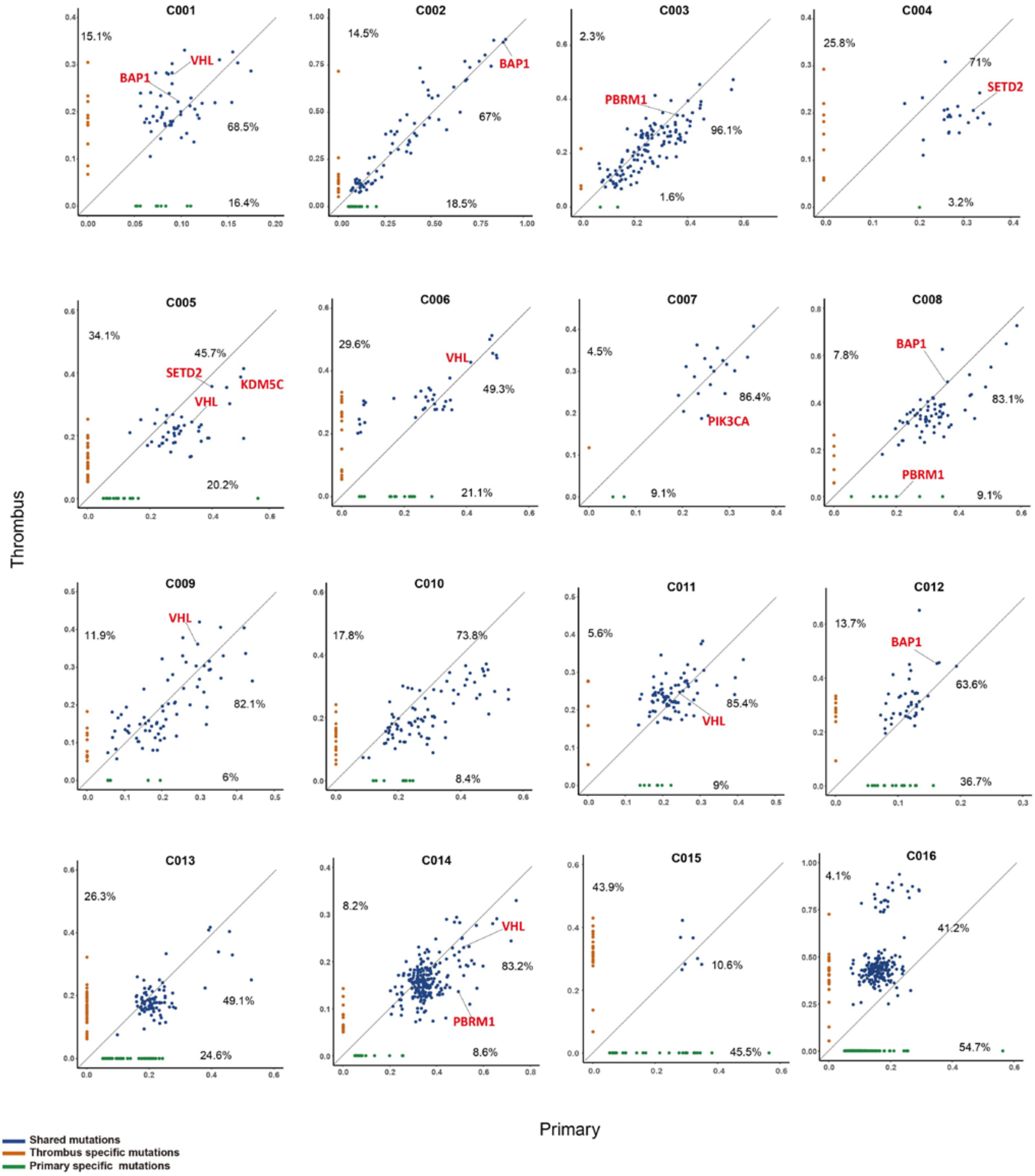
C001
15.1%
VHL
BAP1
68.5%
16.4%
C002
14.5%
BAP1
67%
18.5%
C003
2.3%
PBRM1
96.1%
1.6%
C004
25.8%
71%
SETD2
3.2%
C005
34.1%
45.7%
SETD2
KDM5C
VHL
20.2%
C006
29.6%
VHL
49.3%
21.1%
C007
4.5%
86.4%
PIK3CA
9.1%
C008
7.8%
BAP1
83.1%
PBRM1
9.1%
C009
VHL
11.9%
82.1%
6%
C010
17.8%
73.8%
8.4%
C011
5.6%
85.4%
VHL
9%
C012
13.7%
BAP1
63.6%
36.7%
C013
26.3%
49.1%
24.6%
C014
8.2%
VHL
83.2%
PBRM1
8.6%
C015
43.9%
10.6%
45.5%
C016
4.1%
41.2%
54.7%
Thrombus
Primary
Shared mutations
Thrombus specific mutations
Primary specific mutations

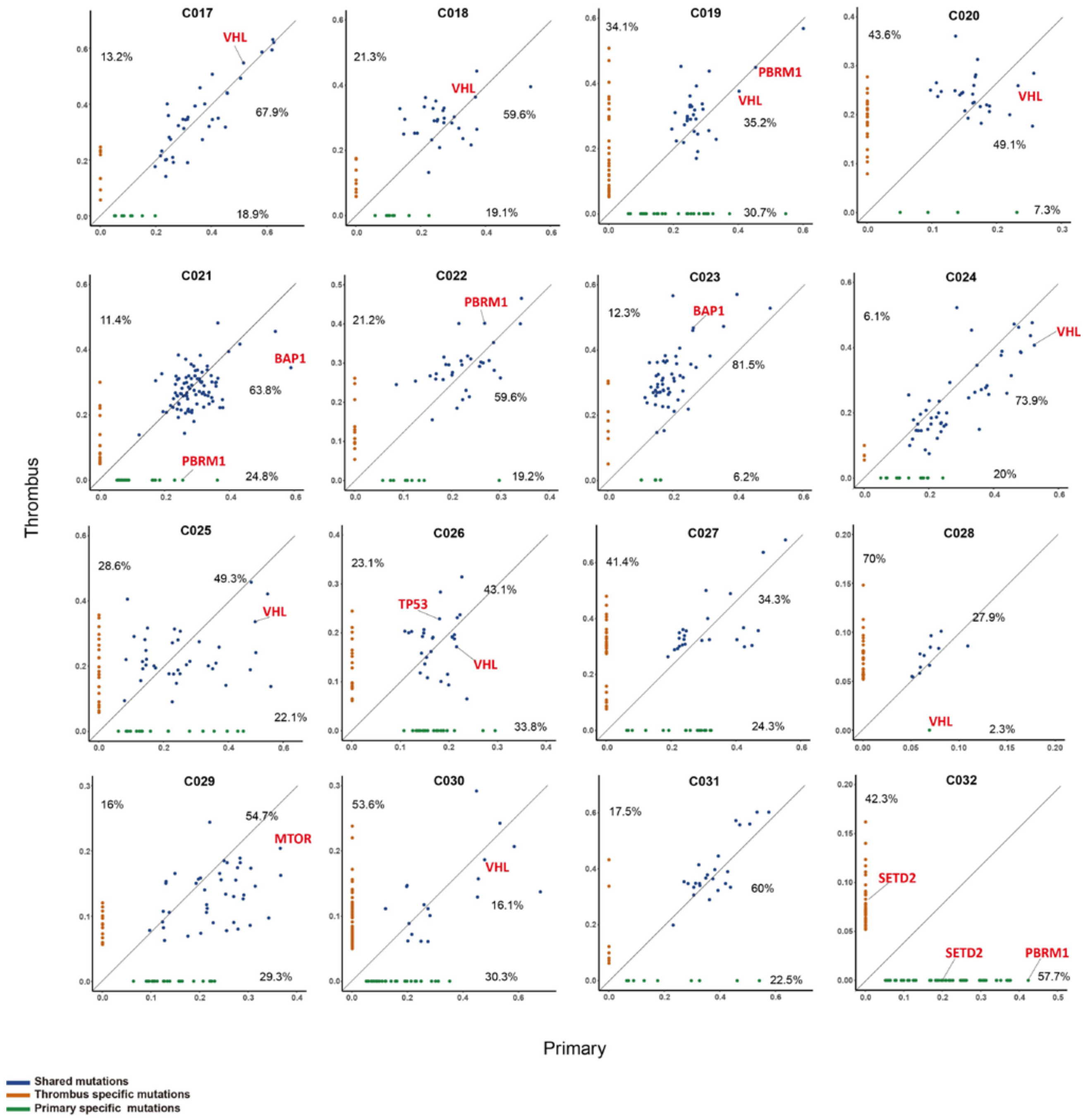

C017
13.2%
VHL
67.9%
18.9%
C018
21.3%
VHL
59.6%
19.1%
C019
34.1%
PBRM1
VHL
35.2%
30.7%
C020
43.6%
VHL
49.1%
7.3%
C021
11.4%
BAP1
63.8%
PBRM1
24.8%
C022
21.2%
PBRM1
59.6%
19.2%
C023
12.3%
BAP1
81.5%
6.2%
C024
6.1%
VHL
73.9%
20%
C025
28.6%
49.3%
VHL
22.1%
C026
23.1%
43.1%
TP53
VHL
33.8%
C027
41.4%
34.3%
24.3%
C028
70%
27.9%
VHL
2.3%
C029
16%
54.7%
MTOR
29.3%
C030
53.6%
VHL
16.1%
30.3%
C031
17.5%
60%
22.5%
C032
42.3%
SETD2
SETD2
PBRM1
57.7%
Thrombus
Primary
Shared mutations
Thrombus specific mutations
Primary specific mutations

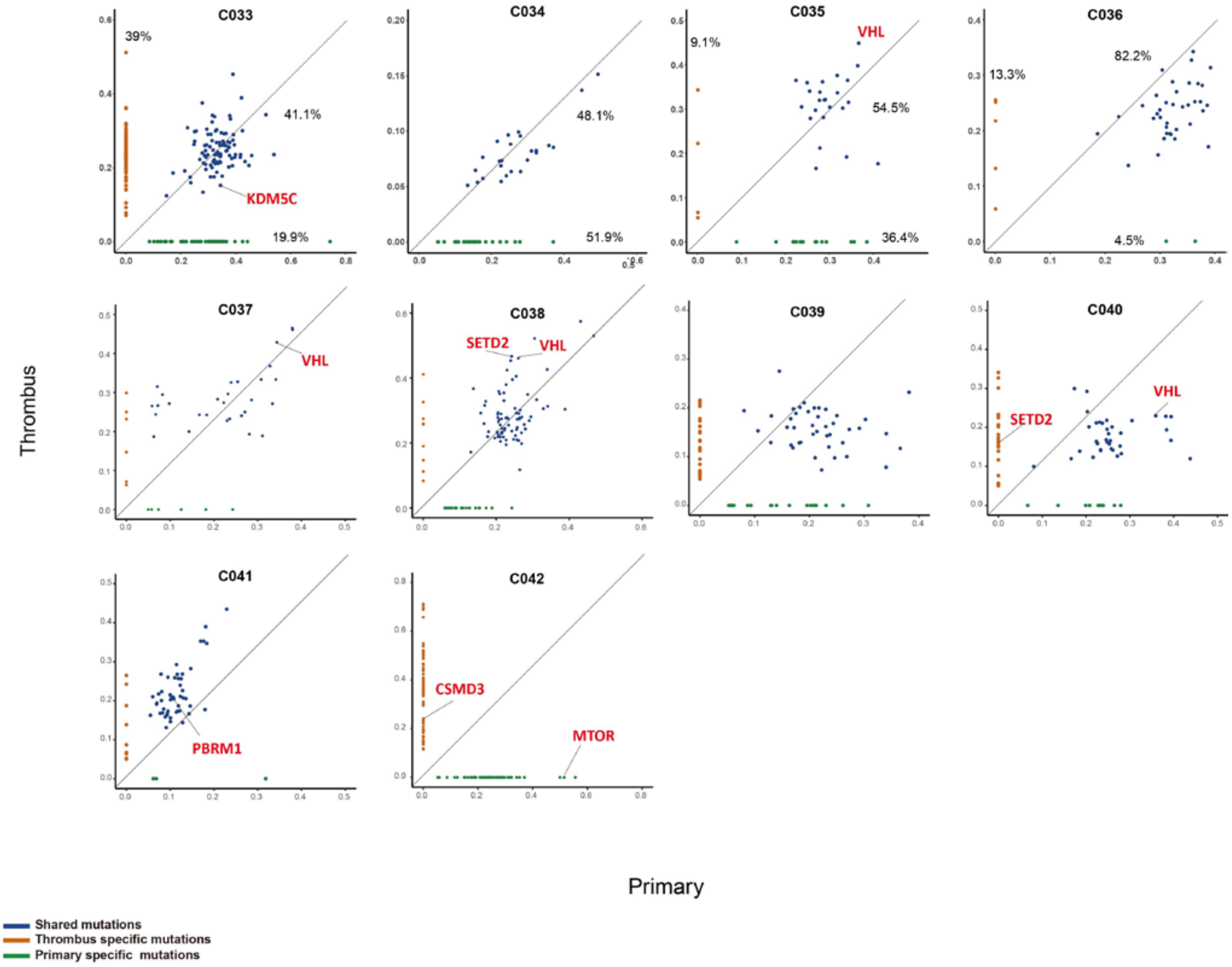

Supplementary Figure 7: Diagonal plots of non-synonymous mutations detected in 42 paired ccRCC specimens. Observed allele frequencies from primary tumors and thrombi are plotted for each mutation. Major driver mutations are indicated.

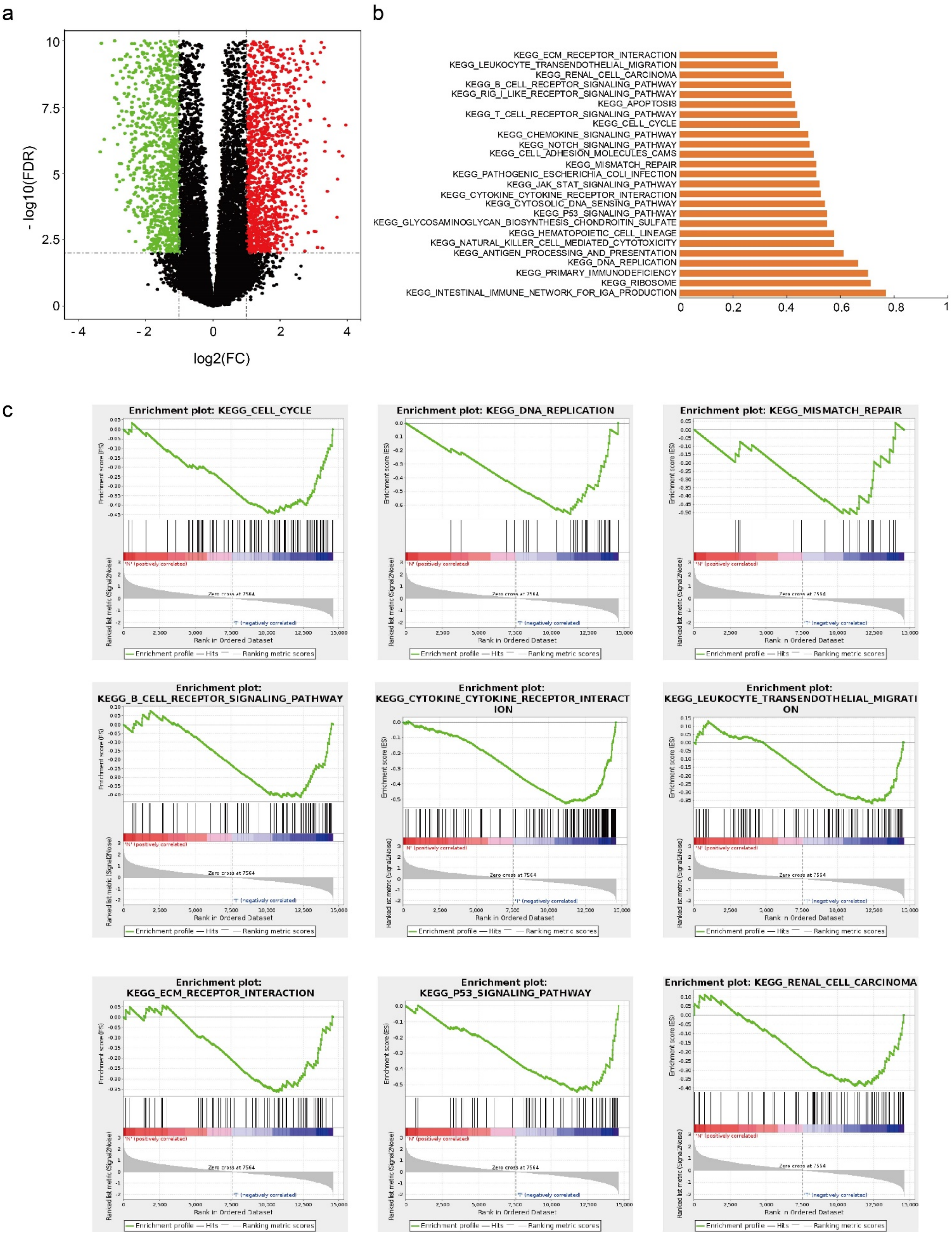

Supplementary Figure 8: Differentially expressed genes and pathways between ccRCC tumors and normal kidney tissues. a, Volcano plot of differentially expressed genes between ccRCC tumors and normal kidney tissues with a threshold fold-change of 2 and FDR < 0.01. **b,** Top 25 pathways enriched in tumor

samples according to GSEA enrichment analysis. **c,** Enrichment plots showed that the immune response and many cancer-associated pathways were activated in the tumor samples.

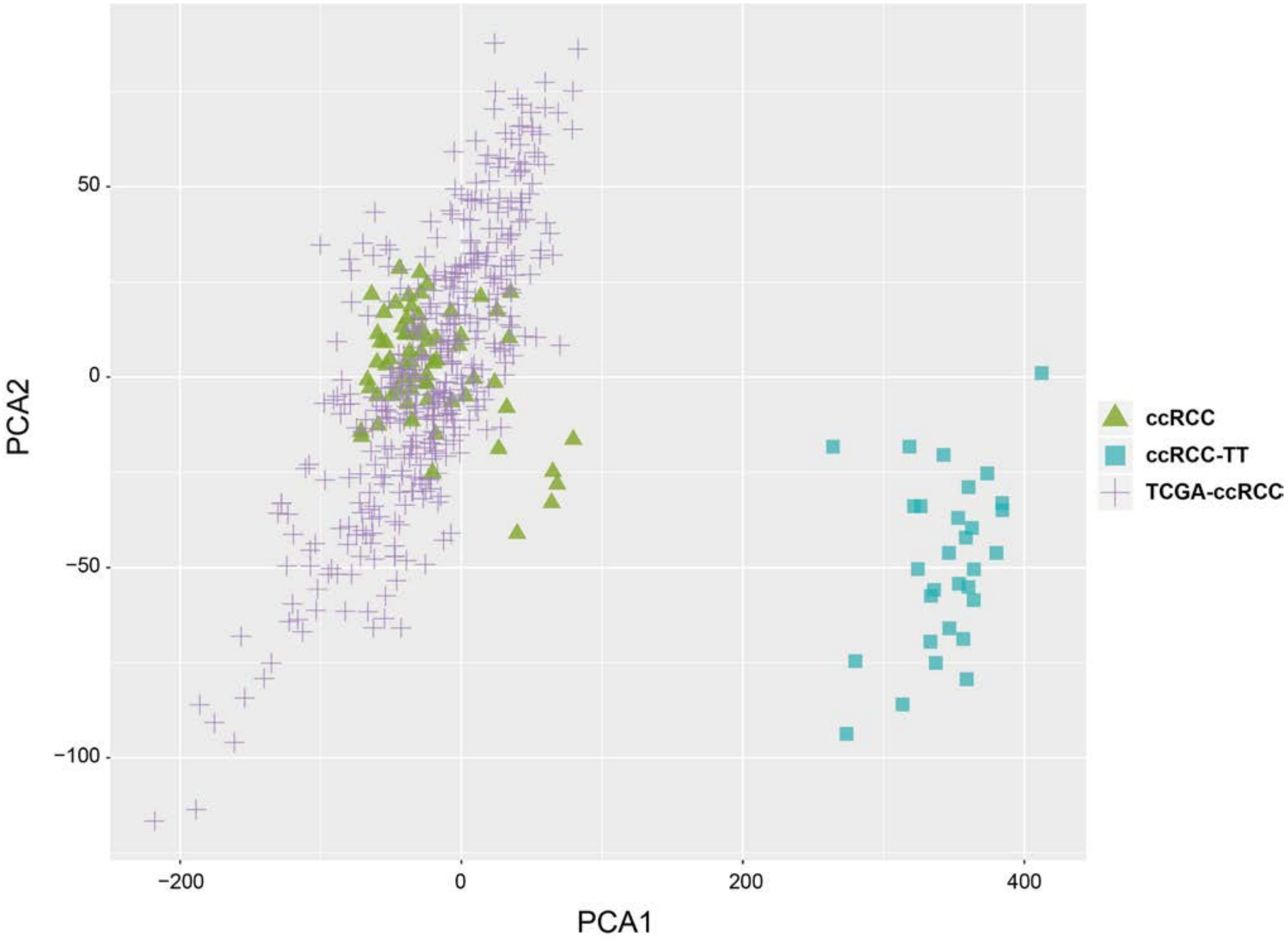

Supplementary Figure 9: PCA plot showing that patients with thrombus were clustered to a group separate from the TCGA cohort and our ccRCC and ccRCC-TT cohorts.

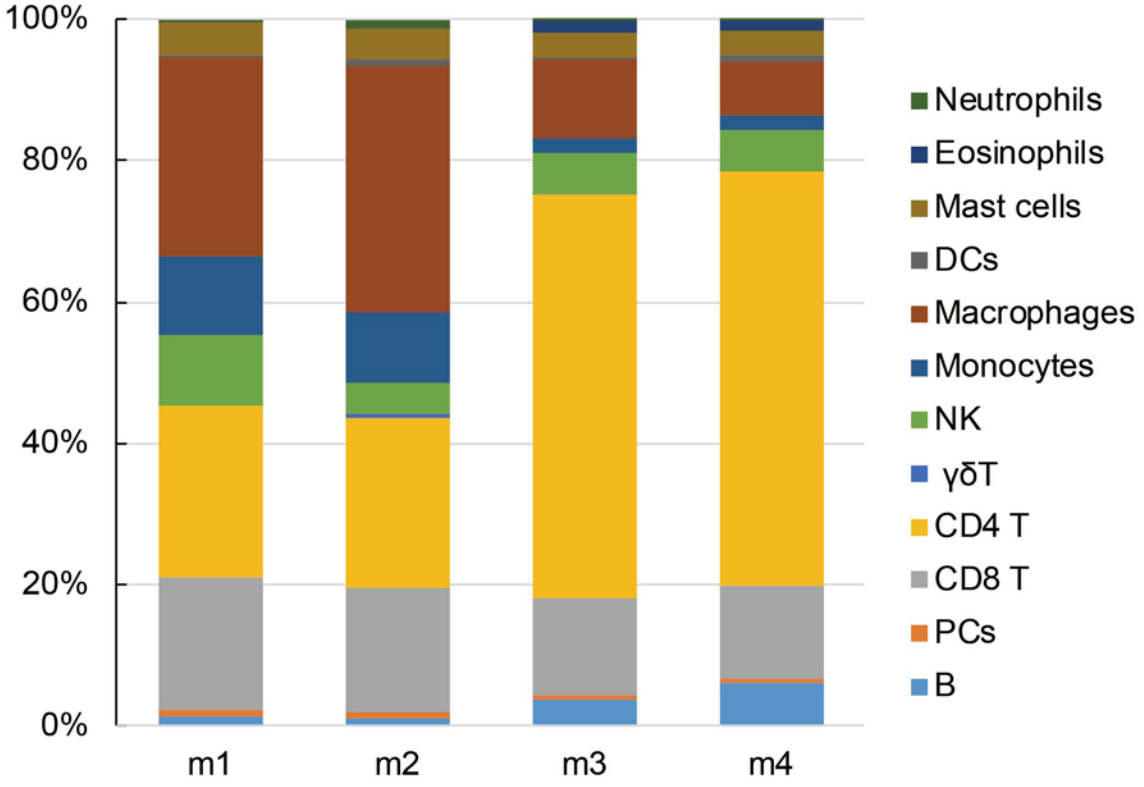

Supplementary Figure 10: Relative leukocyte fractions enumerated in tumors by CIBERSORT. Estimated mRNA fractions of 22 leukocyte subsets across 98 samples, pooled into 12 immune populations here for clarity. The source data underlying Supplementary Fig. 10 is provided as a Source Data file.

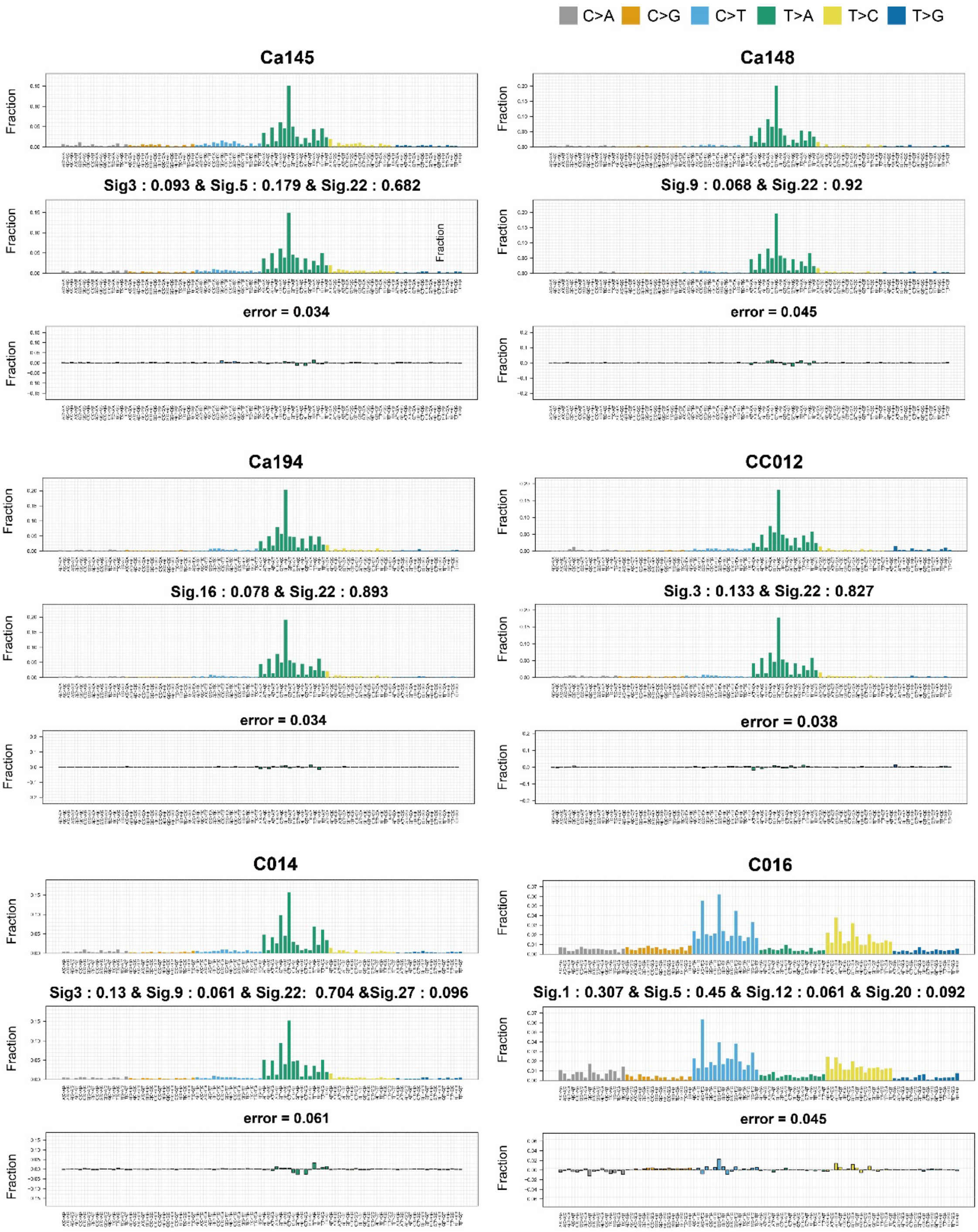

Supplementary Figure 11: The mutational spectra and deconstructed signatures of six patients (Ca145, Ca148, Ca194, CC012, C014 and C016) with a hypermutator phenotype.

	ccRCC	TCGA
**Sample(n)**	152	417
**Age (median; range)**	55.5(25-87)	61(26-90)
**Gender**		
Male	111(73.0%)	271(65.0%)
Female	41(27.0%)	146(35.0%)
**Histology**	Clear cell	Clear cell
**Tumor Size (mm; median)**	50	55
**Staging (TNM)**		
T1	95(62.5%)	202(48.4%)
T2	3(2.0%)	49(11.8%)
T3	48(31.6%)	160(38.4%)
T4	6(3.9%)	6(1.4%)
**Nodes**		
N0	142(93.4%)	191(45.8%)
N1	9(5.9%)	12(2.9%)
Nx	1(0.7%)	214(51.3%)
**Metastasis**		
M0	142(93.4%)	350(83.9%)
M1	11(6.6%)	67(16.1%)

Supplementary Table 1. Summary of clinical information for the study cohorts

		Chinese mRNA cluster	
		**m3**	**m4**
	**T1**	0	24
**TGCA cluster**	**T2**	0	10
	**T3**	15	7
	**T4**	9	2

Supplementary Table 2. Concordance analysis between our RNA subsets and the TCGA RNA subsets. The numbers of samples overlapping between our mRNA-based clusters and previously described TCGA mRNA-based clusters are shown. Red numbers indicate a significant overlap (p < 0.05, chi-squared test).
