# Supplementary material for: Integrative genomic study of Chinese clear cell renal cell carcinoma reveals features associated with thrombus
Source: Nat Commun. 2020 Feb 6;11:739. doi: 10.1038/s41467-020-14601-9 (PMC7005298; doi:10.1038/s41467-020-14601-9)
Supplement: Supplementary file 2 — Description of Additional Supplementary Files [file 41467_2020_14601_MOESM2_ESM.pdf]

Description of Additional Supplementary Files

File Name: Supplementary Data 1
Description: Clinical infromation of all patients

File Name: Supplementary Data 2
Description: Sequencing depth and coverage of samples
