# Supplementary material for: Integrative genomic study of Chinese clear cell renal cell carcinoma reveals features associated with thrombus
Source: Nat Commun. 2020 Feb 6;11:739. doi: 10.1038/s41467-020-14601-9 (PMC7005298; doi:10.1038/s41467-020-14601-9)
Supplement: Supplementary file 5 — Reporting Summary [file 41467_2020_14601_MOESM5_ESM.pdf]

natureresearch

Corresponding author(s): Fan Bai, Di Wu, Lu-lin Ma

Last updated by author(s): Dec 6, 2019

Reporting Summary

Nature Research wishes to improve the reproducibility of the work that we publish. This form provides structure for consistency and transparency in reporting. For further information on Nature Research policies, see Authors & Referees and the Editorial Policy Checklist.

Statistics

For all statistical analyses, confirm that the following items are present in the figure legend, table legend, main text, or Methods section.

n/a	Confirmed	
☐	☒	The exact sample size (n) for each experimental group/condition, given as a discrete number and unit of measurement
☐	☒	A statement on whether measurements were taken from distinct samples or whether the same sample was measured repeatedly
☐	☒	The statistical test(s) used AND whether they are one- or two-sided<br>*Only common tests should be described solely by name; describe more complex techniques in the Methods section.*
☐	☒	A description of all covariates tested
☒	☐	A description of any assumptions or corrections, such as tests of normality and adjustment for multiple comparisons
☐	☒	A full description of the statistical parameters including central tendency (e.g. means) or other basic estimates (e.g. regression coefficient) AND variation (e.g. standard deviation) or associated estimates of uncertainty (e.g. confidence intervals)
☐	☒	For null hypothesis testing, the test statistic (e.g. F, t, r) with confidence intervals, effect sizes, degrees of freedom and P value noted<br>*Give P values as exact values whenever suitable.*
☒	☐	For Bayesian analysis, information on the choice of priors and Markov chain Monte Carlo settings
☒	☐	For hierarchical and complex designs, identification of the appropriate level for tests and full reporting of outcomes
☐	☒	Estimates of effect sizes (e.g. Cohen's d, Pearson's r), indicating how they were calculated

Our web collection on statistics for biologists contains articles on many of the points above.

Software and code

Policy information about availability of computer code

Data collection	No special software was used.
Data analysis	BWA v0.5.9, Samtools v0.1.19, GATK v2.1-8, Picard tools, MuTect, Sequenza R v.2.1.1, SNPEFF v3.0, DeconstructSigs, GSEA, SigProfiler, HTseq, HISAT2, DESeq2, MutSigCV, GISTIC2.

For manuscripts utilizing custom algorithms or software that are central to the research but not yet described in published literature, software must be made available to editors/reviewers. We strongly encourage code deposition in a community repository (e.g. GitHub). See the Nature Research guidelines for submitting code & software for further information.

Data

Policy information about availability of data

All manuscripts must include a data availability statement. This statement should provide the following information, where applicable:

- Accession codes, unique identifiers, or web links for publicly available datasets
- A list of figures that have associated raw data
- A description of any restrictions on data availability

The whole exome and transcriptome sequencing data have been deposited in the Genome Sequence Archive in BIG Data Center, Beijing Institute of Genomics (BIG), Chinese Academy of Sciences, under accession numbers CRA001500 and CRA001952. The source data underlying Figs. 1a-c, 2c, 2f, 3a-c, 4b, 4d, and 5a, as well as Supplementary Figs. 4 and 10 are provided as a Source Data file.

Field-specific reporting

Please select the one below that is the best fit for your research. If you are not sure, read the appropriate sections before making your selection.

☒ Life sciences ☐ Behavioural & social sciences ☐ Ecological, evolutionary & environmental sciences

For a reference copy of the document with all sections, see nature.com/documents/nr-reporting-summary-flat.pdf

Life sciences study design

All studies must disclose on these points even when the disclosure is negative.

Sample size	No statistical methods were used to predetermine sample size.
Data exclusions	There is no data that were excluded from the analyses.
Replication	There is no replication in our study.
Randomization	The experiments were not randomized. Based on our research design, all of samples were grouped into normal samples, primary tumors and thrombi by their biological and histological features.
Blinding	The investigators were not blinded to allocation during experiments and outcome assessment.

Reporting for specific materials, systems and methods

We require information from authors about some types of materials, experimental systems and methods used in many studies. Here, indicate whether each material, system or method listed is relevant to your study. If you are not sure if a list item applies to your research, read the appropriate section before selecting a response.

Materials & experimental systems

n/a	Involved in the study	
☒	☐	Antibodies
☒	☐	Eukaryotic cell lines
☒	☐	Palaeontology
☒	☐	Animals and other organisms
☐	☒	Human research participants
☒	☐	Clinical data

Methods

n/a	Involved in the study	
☒	☐	ChIP-seq
☒	☐	Flow cytometry
☒	☐	MRI-based neuroimaging

Human research participants

Policy information about studies involving human research participants

Population characteristics	This study involved 152 participants, who were diagnosed with clear cell renal cell carcinoma and eceived no treatment before surgery. 73% of participants are male. Their age range is from 25 to 87. Population characteristics in this study are provided in detail in Supplementary table 1.
Recruitment	Participants were included if they had histologically confirmed clear cell renal cell carcinoma and received no treatment before surgery.
Ethics oversight	Peking University Third Hospital and Chinese PLA General Hospital

Note that full information on the approval of the study protocol must also be provided in the manuscript.
